# Association of High Somatic Cell Counts Prior to Dry off to the Incidence of Periparturient Diseases in Holstein Dairy Cows

**DOI:** 10.3390/vetsci9110624

**Published:** 2022-11-09

**Authors:** Ashley Egyedy, Eduardo Barahona Rosales, Burim N. Ametaj

**Affiliations:** Department of Agriculture, Food and Nutritional Science, University of Alberta, Edmonton, AB T6G 2P5, Canada

**Keywords:** dairy cow, somatic cell count, periparturient diseases

## Abstract

**Simple Summary:**

In this study, we looked at a possible link between udder infection in dairy cows one week before drying off and the incidence of several periparturient diseases postpartum, including uterine infection (metritis), lameness, ketosis, and retained placenta. Infection of the mammary gland in dairy cows is associated with a strong host immune response and the transfer of neutrophils known as somatic cells. If the number of somatic cells in the milk is greater than 200,000 cells/mL of milk, the cow has subclinical or clinical mastitis; if the number of SCC is less than 200,000 cells, the cow is considered healthy. According to the findings of this study, cows with a high somatic cell count in their milk before drying off had a higher incidence of ketosis postpartum. Cows with a high somatic cell count were more likely to have a retained placenta, uterine infection, and lameness after calving. Infection of the udder, as indicated by high somatic cell counts in the milk, was also linked to lower milk yield and lower concentrations of milk sugar and protein. Our findings suggest that udder inflammation prior to drying off can make dairy cows more susceptible to other periparturient diseases.

**Abstract:**

Intramammary infections (mastitis) of dairy cows, along with other periparturient diseases, have become problematic within the dairy industry as they lead to loss of milk production. The main objective of this study was to determine whether elevated somatic cell counts (SCC) in cows prior to drying off are related to the incidence of other periparturient diseases. Additionally, we determined whether milk composition and milk yield are affected by a high SCC prior to drying off. Somatic cell counts of milk samples were determined prior to dry off (n = 140) and were used to classify cows in the study as high (>200,000 cells/mL) or low (<200,000 cells/mL) SCC. The composition of milk was analyzed before drying off and at 1 and 2 weeks after calving. The results showed that an elevated SCC before drying off was related to the incidence of ketosis. Cows with a high SCC at drying off also showed an increased likelihood of retained placenta, metritis, and lameness postpartum; however, it was not statistically significant. Milk lactose was lower in cows with high SCC, whereas protein content was lower after parturition. Milk production was lower for cows with pre-drying elevated SCC, particularly for cows with retained placenta, ketosis, and mastitis. In conclusion, cows with pre-drying elevated SCC were more likely to develop disease after parturition and produce less milk and with lower lactose and protein content.

## 1. Introduction

Intramammary infection (IMI) of dairy cows, known as mastitis, is defined as inflammation of the mammary gland due to bacterial infection or other infective agents. Infection of the udder can cause subclinical mastitis or clinical mastitis. Clinical signs of an IMI include increased SCC, decreased milk yield, altered milk appearance and composition, as well as swelling, heat, and pain of the udder [1]. About 50% of cows in a dairy herd may have subclinical IMI [2]. A mastitis infection can lead to a significant cost of $662 per milking cow per year in a dairy farm in Canada [3]. Elevation of milk SCC is the most widely used and effective method for determining IMI in dairy cows. Somatic cells in milk consist of leukocytes, mainly macrophages and neutrophils [4]. It has been suggested that SCC in healthy mammary glands is less than 100,000 cells/mL and counts greater than that indicate presence of infection [5]. In Canada, the presence of an IMI is indicated when SCC exceeds 200,000 cells/mL [6]. Losses in milk production can be significantly affected by IMI within a dairy herd. The decrease in milk production during infection is the result of increased permeability of the blood–milk barrier, damage to the mammary tissue [7], and decreased lactose synthesis [8]. Additionally, the decline in milk production occurs during other diseases including uterine infections [9], retained placenta [10], ruminal acidosis [11], lameness [12], and ketosis [13].

Milk composition also changes during an IMI. The decline in milk fat can be attributed to neutrophils mistakenly engulfing milk fat globules during phagocytosis [14], and oxidation by lipase enzymes from leukocytes [8,14,15].

Lactose is the main carbohydrate of bovine milk and functions as an osmotic regulator for milk volume. Lactose is synthesized from one galactose molecule, which is derived from glucose absorbed from the blood, and one glucose molecule also absorbed from the blood [16]. The decrease in lactose in the milk may be related to a decrease in blood glucose. Lactose concentrations decrease during IMI due to the greater gap between the tight junctions through which lactose enters the bloodstream, which has been confirmed by several authors [15]. 

Mastitis has been associated with decreased synthesis of milk protein, including casein and whey proteins, including α-lactalbumin [8]. Conversely, the concentration of serum albumin, immunoglobulins, and lactoferrin increases during IMI as a part of the immune response [7,8,15]. 

Pregnant lactating dairy cows enter a dry period when the mammary glands stop producing milk about 8 weeks before the next parturition. The dry period is beneficial for dairy cows to maximize milk production in the next lactation cycle [17]. However, the incidence of new IMI in dairy cows was found to be highest at the start of the dry period and before parturition [18]. Current dry treatment protocols, including the use of antibiotics and teat sealant, have been shown to lower the incidence rate of IMI during the dry period by 7.3% [19]. However, dairy cows undergo various metabolic changes as parturition approaches, associated with immunosuppression and increased susceptibility to periparturient disease [20,21,22]. 

Common periparturient diseases that occur in dairy cows postpartum include ketosis, retained placenta, metritis, mastitis, and lameness. Ketosis is characterized by an increase in ketone bodies (e.g., β-hydroxybutyric acid) in the urine, blood, and milk [12]. Retained placenta is characterized by the inability to expel fetal membranes within 24 h of calving. Metritis is a bacterial infection of the uterus that results in inflammation of the uterine tract [23]. Lameness is characterized by inflammation of the corium and germinal layer accompanied with overt signs including abnormal gait and hoof problems [24]. Archer et al. [25] reported a negative relationship between SCC and locomotion scoring in cattle. 

Research conducted by our laboratory revealed that the SCC was elevated during the week of diagnosis of several periparturient diseases. The results showed a significant increase in SCC during diagnosis of several periparturient diseases, but the SCC were within the normal ranges, except for subclinical mastitis where SCC were >200,000 cells/mL [26]. It has been suggested that bacterial endotoxins may play a role in the etiopathology of multiple periparturient diseases [27,28] and it has been reported that endotoxins can translocate from the mammary gland to the systemic circulation [29,30]. 

We hypothesized that cows with high SCC prior to drying off may be more susceptible to the incidence of various periparturient diseases; have changes in milk composition and produce less milk than their healthy counterparts. Therefore, the primary objectives of this study were to determine whether cows with a SCC higher than 200,000 cells/mL (indication of subclinical mammary infection) prior to dry-off are associated with a higher incidence of periparturient disease during the first 2 weeks after calving. In addition, the relationships between SCC and changes in milk composition prior to drying off and during the first 2 weeks after parturition were evaluated; and whether high SCC is related to lower milk production.

## 2. Materials and Methods

### 2.1. Animals and Experimental Design

The study was conducted at 2 dairy farms located in Edmonton and Ponoka, Alberta, Canada. A total of 140 pregnant Holstein dairy cows consisting of 82 multiparous and 58 primiparous were used for this study. The number of cows used per dairy farm was 104 and 36 cows in Edmonton and Ponoka, respectively. The University of Alberta dairy farm in Edmonton uses a tie-stall system and the conventional farm in Ponoka uses a free-stall parlor system. All experimental procedures were approved by the University of Alberta’s Animal Care and Use Committee for Livestock. Proper care of each animal was followed in accordance with the guidelines of the Canadian Council on Animal Care [31]. 

The cows used in the study were randomly selected (heifers were excluded) and had to meet the following criteria: (1) pregnant and (2) entering the dry period. Each cow was sampled approximately one week prior to drying off, then resampled at 1 and 2 weeks postpartum. Milk samples were taken at all time points, and approximately 20 mL was transferred to a DHI (CanWest Dairy Herd Improvement, Edmonton, AB, Canada) vial for laboratory analysis. The rest of the milk sample were stored at −20 °C until further analyses. The 20 mL DHI milk vial was shipped to the Central Milk Testing Lab located in Edmonton, AB, Canada (CanWest DHI, Canada) and analyzed by mid-infrared spectroscopy (MilkoScan 605; A/S Foss Electric, Hillerød, Denmark). Milk samples were analyzed for concentrations of fat, protein, lactose, milk urea nitrogen (MUN), total solids (TS), and somatic cell counts (SCC). The fat-to-protein ratios (FPR) were calculated by dividing total fat% by the total protein% per cow. 

Fecal scores were determined per fecal sample on a scale of 1 to 5 where a score of 1 = diarrhea; 2 = appears runny and does not form a distinct pile 3 = optimal score, porridge-like appearance, will stack up at 4–5 cm; 4 = manure is thicker and will stick to shoe, accumulates up to more than 5 cm; 5 = solid fecal balls [32]. 

Body condition scoring was performed for all animals at each sampling time point in accordance with the Elanco body condition scoring in dairy cattle on a scale of 1 to 5 at 0.25-point intervals. Additional information including lactation, age, previous 305 days of milk yield, and date of the drying off were recorded. The duration of the drying period (days) was determined for each animal using DairyComp software (Lactanet, Guelph, ON, Canada). Estimated calving dates and actual calving dates were recorded and whether calving was earlier or later than the expected calving date were recorded. Daily milk weights (kg) were recorded up to the first 60 days in milk (DIM). 

At 60+/− 3 days before the expected day of parturition or when the milk production was below 11 kg/d cows were abruptly dried off. After the final milking each teat was cleaned with an individual alcohol wipe. Then, each quarter was infused with 10 mL of dry cow therapy, Cefa-Dri (Boehringer Ingelheim Ltd., Burlington, ON, Canada). Immediately after Cefa-Dri treatment OrbeSeal (Zoetis, Canada Inc., Kirkland, QC, Canada) was infused. Then, each teat was dipped into a teat dip solution. Then, animals were relocated to an outside pen. 

### 2.2. Clinical Observations for Periparturient Disease, Grouping Based on SCC and Disease Diagnosis

The health status of each cow was monitored daily for clinical signs of disease by trained staff members. All periparturient diseases and treatments were recorded during the experimental period. Breeding and culling records were recorded during the first 6 weeks after calving. Animals were removed from the study if death/culling occurred during the dry period or after calving where no samples could be obtained. External signs were observed including alertness, appetite, fecal consistency, and body condition score (BCS). 

Postpartum mastitis was determined by SCC being >200,000 cells/mL. Retained placenta was diagnosed based on cows failing to expel fetal membranes within 24 h of calving. Metritis was assessed using vaginal mucus score by the Metricheck device (Simcro, New Zealand). The device was disinfected with Nolvasan (Zoetis, Kalamazoo, MI, USA) containing 2% chlorhexidine diacetate and ethanol. The cows’ external genitalia were disinfected with a 16% iodine solution (Vetoquinol N.-A, Inc., Lavaltrie, QC, Canada), then Metricheck was inserted into the reproductive tract to obtain vaginal mucus. Mucus assessment was performed according to Sheldon et al., (2006) where 0 = clear or translucent mucus; 1 = mucus containing flecks of white or whitish pus; 2 = discharge with ≤50% puss or whitish mucopurulent material; 3 = discharge containing ≥50% purulent material, typically white or yellow or bloody. Metritis was diagnosed when the mucus score was 3. 

Lameness was diagnosed using a locomotion scoring system following farm standard operating procedures [33]. Locomotion scoring was assessed according to the Zinpro guidelines (adopted from [33]) for evaluation of dairy cattle locomotion on a scale of 1 to 5, where 1 = normal; 2 = slightly lame; 3 = moderately lame; 4 = lame; and 5 = severely lame. 

Diagnosis of ketosis was made using a KetoStix strip (Bayer Corp. Diagnostics Division, Tarrytown, NY, USA) which detected urinary acetoacetate (AcAc) and clinical signs such as loss of appetite, poor body condition, and treatment in accordance with standard farm operating procedures. The measurement of AcAc in urine was based on the color intensity found on the KetoStix package and was scored using KetoStix strips in five categories where negative = 0 mmol/L; trace = 0.5 mmol/L; small = 1.5 mmol/L; moderate = 4 mmol/L; Large = 8 mmol/L of AcAc.

**Figure 1:**
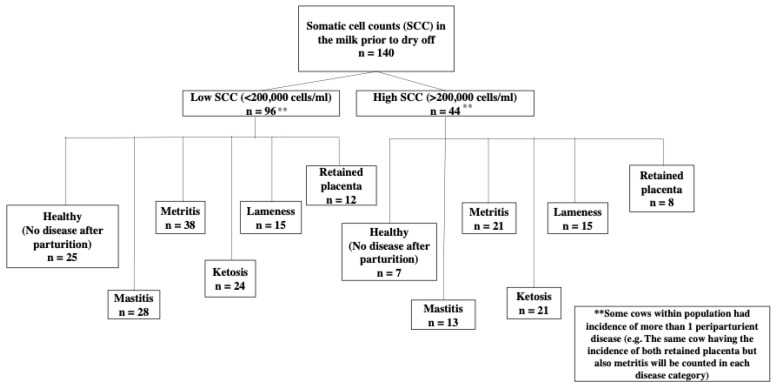
Flow diagram for the incidence rate of diseases for cows grouped based on somatic cell counts (SCC) in the milk obtained at prior to dry off (approximately 1 week before the expected date of dry off). Cows with SCC < 200,000 cells/mL were sorted into the Low SCC group, and cows with SCC > 200,000 cells/mL were sorted into the High SCC group. After parturition, cows from each group were evaluated for each of the five diseases of interest (i.e., mastitis, metritis, ketosis, lameness, and retained placenta) with the total number of cows per each disease indicated in each box in the diagram. Healthy cows were identified if no incidence of disease occurred.

The experimental design for this study was a nested case–control design (Figure 1) in which SCC in the milk prior to drying was used to divide cows into two groups: (1) low SCC (<200,000 cells/mL) and (2) elevated SCC (>200,000 cells/mL). Cows were evaluated for the incidence of five periparturient diseases including mastitis, metritis, lameness, retained placenta, and ketosis. The cows were diagnosed with mastitis according to the farm’s standard operating procedures. 

For comparisons among groups for analysis, cows diagnosed with postpartum disease were labeled as low-disease (LD) and high-disease (HD) cows based on the SCC prior to drying and whether one of the five diseases was diagnosed (e.g., cow with >200,000 cells/mL prior to drying off and diagnosed with metritis after parturition was considered a high-disease animal). Healthy cows were identified if their SCC were <200,000 cells/mL, with no incidence of disease during the experimental period. Healthy cows were used as a comparison with the diseased groups and were classified as a healthy group (HG). 

### 2.3. Statistical Analysis

In this study, cows were blocked by the SCC in the milk that was determined prior to dry off and were assigned a low SCC group and a high SCC group. Cows with SCC < 200,000 cells/mL were classified as low and cows with SCC > 200,000 cells/mL were classified as high. A further blocking was performed on the basis that the cows had been diagnosed with postpartum disease and were classified as LD and HD. The study is a nested case–control design in which cows were blocked into two groups based on the SCC determined prior to drying off, and then further blocked based on the diagnosis of periparturient disease. We performed a separate analysis for each disease. 

Binary data for disease incidence were analyzed using the crosstabs function in MedCalc 18.2.1 software [34]. The odds ratio was calculated for the entire population using the crosstabs function to determine the likelihood of a high SCC cow’s incidence of post-partum disease compared to low SCC. 

The frequency for cows diagnosed with disease was calculated using the FREQ procedure in SAS 9.2 software (SAS Institute Inc., Cary, NC, USA). The frequency was calculated for the number of cows diagnosed with disease with low SCC and high SCC, and free of disease (healthy group). Additionally, the frequency of cows with either single or multiple diseases after parturition was calculated. The percentages were calculated out of the total population (n = 140).

For the control group, we chose 15 healthy cows with no signs of illness, with similar BCS, fecal score, milk composition, and dry period length, and whose SCC was consistently <200,000 cells/mL of milk throughout the study. This control group was categorized as the healthy group (HG) and was used to compare with each disease of interest. We randomly selected seven or eight cows from each SCC group (low or high) diagnosed with postpartum disease and classified them as Low-Disease (LD; <200,000, cells/mL of milk) and seven or eight cows from the high SCC group as High-Disease (HD; >200,000 cells/mL of milk). During the study period, animals from the diseased groups (LD or HD) had to be diagnosed with one disease. For example, one cow was diagnosed with metritis but had no other diseases. However, in order to have a sufficient number of sick animals for comparison, we had to select cows with multiple diseases. As a result, when fitting the statistical model, the number of diseases per cow was taken into account. The metritis group was the exception; all cows were diagnosed only with metritis during the study, so the number of diseases was excluded from the statistical model for metritis. 

Data on milk composition and milk production were analyzed with SAS 9.2. The normality of the data was first verified using the UNIVARIATE procedure for each group and variable; however, the data did not follow a normal distribution. We then used the GLIMMIX repeated measures procedure for non-normal distribution data. The farm and cow effect were considered a random effect in the model statement. The covariance structure was modeled according to the smaller Akaike information criterion (AIC) and the generated Bayesian information criterion (BIC) values. The health status effect was forced into the model statement because we wanted to determine if changes in milk composition and production differed among groups. Therefore, our main model was the following:

Yijkl = μ + Hi + eijkl

where μ = the overall population mean; Hi = the fixed effect of health status i (i = 1–3, healthy cows compared to LD and HD groups separately), and eijkl = the residual error. Additional model fixed effects for week, parity, and number of diseases were examined, along with their corresponding interactions for each milk component by disease group. A backward elimination from a saturated model was performed if the effect was not significant on the response variable. There was no significance in the three- and four-way interactions for all components of milk and FPR for disease, and they were removed from the statistical model.

For the analysis of milk production data, total milk production was calculated and compared with the previous 305 DIM productions among the groups. The data were initially tested for normality using the UNIVARIATE procedure and it was found that they did not follow a normal distribution. All milk yield data were then analyzed using the GLIMMIX procedure. The total milk production model for the first 60 DIM included the effect of health status, previous production of 305 DIM, and the interaction among health status and previous production. The interaction effect was significant only for mastitis and was removed from the statistical model for metritis, retained placenta, ketosis, and lameness. The effect of previous production was significant for metritis, mastitis, and ketosis and was therefore retained in the statistical model. Previous milk yield was not significant for retained placenta and lameness and was therefore not included in the model. The mean and SEM for milk production during the 60 DIM was calculated for each group for each disease. For all data, significance was declared at *p* < 0.05 and tendency at 0.05 ≤ *p* ≤ 0.10. 

## 3. Results

### 3.1. Frequency of Cows Diagnosed with Post-Partum Disease

**Figure 2:**
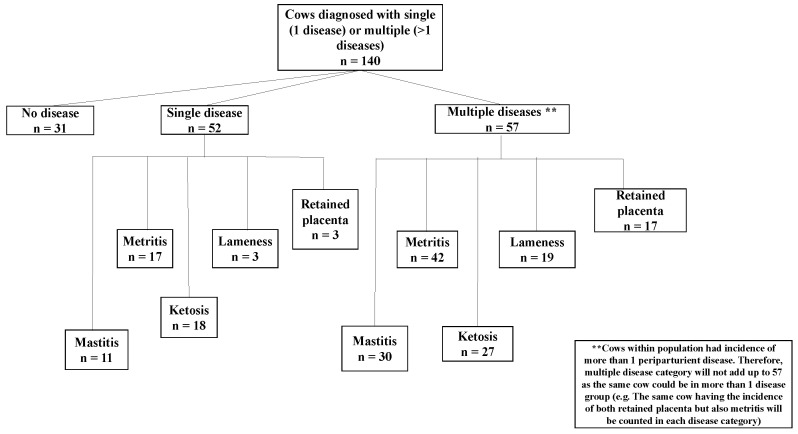
Flow chart of incidence rate of each of five periparturient diseases (i.e., mastitis, metritis, ketosis, lameness, retained placenta) in cows diagnosed with single disease (one disease only), multiple diseases (two diseases or more) and no diseases. Note: Cows may have been diagnosed with one or more postpartum diseases (i.e., the same cow could have been classified as retained placenta and metritis).

The frequency for periparturient disease incidence for SCC groups, parity, and number of diseases is shown in Table 1 and Figure 1. The incidence of cows with single or multiple diseases is shown in Figure 2. Approximately 22.14% (n = 31) of the total population had no postpartum disease. Of the 22.14% healthy cows, roughly 17.14% (n = 24) had a low SCC and were identified as healthy cows, and 5.0% (n = 7) a high SCC before drying off. 

Mastitis was diagnosed in 29.29% (n = 41) of the overall population, with 20.00% (n = 28) having low SCC and 9.29% (n = 13) having high SCC before drying; Mastitis alone was diagnosed in 7.86% (n = 11), and 21.43% (n = 30) had multiple diseases in addition to mastitis. 

The overall population diagnosed with metritis was 42.14% (n = 59), with 27.14% (n = 38) having a low SCC and 15.00% (n = 21) with a high SCC before drying; 12.14% (n = 17) were diagnosed with metritis alone, and 30.00% (n = 42) were diagnosed with multiple diseases, including metritis. 

Lameness was diagnosed in 15.71% (n = 22) of the total population, with 10.71% (n = 15) having low SCC and 5.00% (n = 7) having high SCC prior to dry-off; 2.14% (n = 3) were diagnosed with lameness alone, and 13.57% (n = 19) were diagnosed with multiple diseases in addition to lameness. 

In the retained placenta, 14.29% (n = 20) of the total population were diagnosed with the disease, with 8.57% (n = 12) having low SCC and 5.71% (n = 8) having high SCC before drying off. Only 2.14% (n = 3) were diagnosed with retained placenta only and 12.14% (n = 17) had multiple diseases in addition to retained placentas. 

For ketosis, 32.14% (n = 45) of the total population were diagnosed with the disease, with 17.14% (n = 24) having low SCC, 15.00% (n = 21) having high SCC prior to dry-off; 12.86% (n = 18) were diagnosed with ketosis alone, and 19.29% (n = 27) were diagnosed with multiple diseases, besides ketosis. 

### 3.2. Odds Ratio for the Incidence of Disease for Dairy Cows

To determine whether cows with high SCC prior to being dried off were related to disease incidence, we calculated odds ratios for the likelihood that cows with high SCC versus low SCC before drying off will be diagnosed with postpartum disease of the total population (n = 140). The data are presented in Table 2. 

The overall data showed that cows with elevated SCC before dry-off were more likely to develop ketosis after calving (Table 2). Indeed, the likelihood that cows with a high SCC would develop ketosis was 166% (or 2.66 odds ratio) higher than cows with a low SCC (*p* = 0.01). Although the odds ratio for cows with elevated SCC before drying off and the incidence of metritis, retained placenta, and lameness were not statistically significant, cows with elevated SCC showed a higher risk of postpartum disease incidence. For example, cows with high SCC before drying off had a 43.0% (1.43), 56.0% (1.56), and 31.0% (1.31) increased likelihood to be affected by metritis, retained placenta, and lameness, respectively. In comparison to cows with low SCC prior to dry-off, the odds ratio was less than 1 for all five diseases indicating incidence of an event to occur is less likely due to SCC and other factors may be involved. 

The likelihood of cows being diagnosed with postpartum mastitis was not statistically significant between groups, where both cows with low and high SCC before drying were shown to have increased odds of 2.0%. 

### 3.3. Somatic Cell Counts

Data on the composition of milk among cows of the healthy group (HG), LD, and HD cows for diseases prior to dry off as well as at 1 week and 2 weeks can be found in Table 3, Table 4 and Table 5, respectively (See also Graphs in Supplementary Materials). Mean SCC (multiplied by 10^3^ cells/mL) prior to dry off among all diseases showed that the HD group was significantly greater than the LD and HG (*p* < 0.01) (Table 3). 

This was expected, as we grouped cows prior to drying off based on the concentration of SCC in the milk. Comparison of 1 week means for retained placenta showed a tendency in the HD group with a mean of 1359.86 ± 578.00, compared with 54.07 ± 78.73 and 41.57 ± 101.06 (*p* = 0.06) in the HG and LD group, respectively (Table 5). Similarly, at 2 weeks cows in the HD group had a significantly higher SCC of 223.86 ± 73.47 compared to the LD and HG of 43.57 ± 32.41 and 37.58 ± 22.99, respectively (*p* = 0.02) (Table 5). 

Interestingly, cows with diagnosed mastitis showed a higher average of 939.37 ± 411.81 in the LD group compared to the HD group of 666.50 ± 346.88 at 2 weeks postpartum, although not statistically significant (*p* = 0.23) (Table 4). At 1 week, cows showed increased SCC in the LD group (1993.63 ± 798.24) and the HD group (1396.00 ± 667.96), compared to healthy cows (54.07 ± 96.00) (*p* = 0.15) (Table 4). The number of SCC in cows diagnosed with metritis, ketosis, and lameness was not significant at 1 week (*p* = 0.99; *p* = 0.40; *p* = 0.47, respectively). 

### 3.4. Lactose

Similar to the number of pre-drying SCC discussed in the previous section, lactose concentrations of pre-drying milk were found to be significantly lower (*p* < 0.05) in all disease groups, with the HD group having the lowest lactose concentration (Table 3; (See also Graphs in Supplementary Materials). Cows diagnosed with metritis showed decreased lactose concentrations in the HD group at all three time points (*p* = 0.01). Comparison of pre-drying means showed that the HD cows in the ketosis group had the lowest lactose concentration of 3.73 ± 0.15 compared to the other HD groups of metritis, retained placenta, lameness, and mastitis showing similar means (Table 2 and Table 3). At 1 week, the concentration of lactose for the ketosis group showed that the HD group was still numerically lower than the concentrations for the HG and LD groups of cows (*p* = 0.25) and had a tendency at 2 weeks (*p* = 0.09) (Table 4 and Table 5, See also Supplementary Materials). There was also a tendency in the HD retained placenta, where cows with high SCC had an average lactose concentration before drying of 3.96 ± 0.17 at 1 week compared to 4.31 ± 0.18 and 4.44 ± 0.12 in the LD and HG groups (*p* = 0.10) (Table 3). At 2 weeks, there were no significant differences in lactose concentration for cows with retained placenta (*p* = 0.42) (Table 5). Cows in the mastitis group with high SCC before drying off had the lowest lactose concentration at all three time points where pre-drying time period and 1 week were significantly lower (*p* < 0.01) (Table 3). At 2 weeks, the HD group of mastitis cows showed a lower lactose concentration of 4.32 ± 0.12 compared to 4.60 ± 0.13 in the LD group and 4.55 ± 0.10 in the HG, but the difference did not reach significance (*p* = 0.25) (Table 5). There were no significant differences in lactose concentration at 1 and 2 weeks in the lameness group among the HG, LD, and HD cows, although the HD group consistently had the lowest mean.

### 3.5. Protein

Comparisons of mean milk protein concentrations were significant at all three time points for lameness (*p* < 0.01). Protein concentrations for lameness were found to be significant at all three time points (*p* < 0.01) (Table 3, Table 4 and Table 5; See also Graphs in Supplementary Materials). Interestingly, the HD group had the highest mean protein concentration before drying off (4.10 ± 0.15), whereas the HG and LD groups were lower (3.41 ± 0.10 and 3.79 ± 0.14) (Table 3). After parturition, protein concentration decreased in the HD group compared to HG and LD (3.29 ± 0.12 vs. 3.70 ± 0.10 vs. 3.87 ± 0.13) (Table 2, Table 3 and Table 4), and further decreased at 2 weeks for the HD group (2.93 ± 0.10 vs. 3.38 ± 0.09 vs. 3.39 ± 0.11) (Table 5). 

Similar to lameness, cows with a diagnosed retained placenta in the HD group showed a tendency at prior to dry-off and had a higher mean protein concentration compared to LD and HG cows (3.91 ± 0.18 vs. 3.73 ± 0.14 vs. 3.40 ± 0.11) (Table 3). At 1 week, the mean protein concentration was comparable across all three groups (*p* = 0.97) (Table 4), whereas at 2 weeks the HD group had the lowest numerical mean protein concentration (3.09 ± 0.15) but was not significant (*p* = 0.27) (Table 5). 

Ketosis showed no significant differences in the mean protein concentration among the groups prior to dry off (*p* = 0.55) (Table 3). However, the HD group showed higher mean protein concentrations before drying off of 3.64 ± 0.12 compared to the healthy group of 3.49 ± 0.08 and the LD group of 3.55 ± 0.11 (Table 3). At 1 week, the HD group showed a significantly lower mean protein concentration (3.26 ± 0.11) (Table 4) and continued to decrease at 2 weeks (2.80 ± 0.10) (*p* < 0.01) (Table 5). 

There was no statistical significance for milk protein concentration for metritis and mastitis for all three time points. 

### 3.6. Fat, Fat to Protein Ratio, Milk Urea Nitrogen, and Total Solids

The mean concentrations of fat, FPR, MUN, and TS are shown in Table 3, Table 4 and Table 5. The overall fat concentration in the metritis group prior to dry off showed a tendency with the HD group having the highest mean fat concentration of 6.52 ± 0.96 compared to the HG and LD groups of 4.13 ± 0.56 and 4.79 ± 0.82, respectively (*p* = 0.09) (Table 3). Mean fat concentrations did not show statistical significance at 1 (*p* = 0.60) and 2 weeks (*p* = 0.26) for the metritis group (Table 4 and Table 5, respectively). 

Fat content was not different among the three groups among retained placenta, ketosis, lameness, and mastitis for all three time points. The FPR showed no statistical significance, except for the ketosis group at 2 weeks (*p* < 0.01), where the HD group showed the highest FPR of 1.71 ± 0.19 compared to the HG and LD groups of 1.02 ± 0.10 and 1.21 ± 0.15, respectively (Table 5). Furthermore, MUN and TS concentrations were not significant among the groups for all diseases analyzed for each of the sampling times. 

### 3.7. Milk Production

Milk production data (kg) are presented as total yield for 60 DIM in Table 6. Overall comparisons of average total milk production showed that cows in the HD group had lower milk production than those in the LD and HG groups. Pre-drying high SCC diagnosed with retained placenta had an average total milk yield of 2042.99 ± 216.57 compared to low SCC cows of 2285.54 ± 216.57 and HG of 2716.81 ± 153.14 (*p* = 0.04). 

Cows in the HD group diagnosed with mastitis had a mean total yield of 1970.88 ± 177.47 compared to the LD group of 2652.61 ± 158.63 and the HG of 2742.51 ± 122.88 (*p* < 0.01). The effect of previous 305 DIM yields as well as the interaction among health status and previous yield showed significance for mastitis (*p* < 0.01). The high SCC cows diagnosed with ketosis had a mean yield of 2301.81 ± 152.20 compared to the LD group of 2672.33 ± 139.34 and the HG of 2789.97 ± 107.43 (*p* = 0.05). 

The effect of previous 305-DIM yields was also significant for ketosis (*p* < 0.01). The HD lameness group had a mean yield of 2528.36 ± 122.83 compared to the LD group of 2633.04 ± 122.83 and HG of 2716.81 ± 86.85 but was not statistically significant (*p* = 0.46). Comparisons of means between total milk yields for metritis were not statistically significant (*p* = 0.84), however, the HD group had a lower mean yield (2683.27 ± 93.00) compared to the LD group (2748.07 ± 89.65) and healthy cows (2748.21 ± 68.90). The effect of previous 305 DIM yield was significant on total post calving yield (*p* = 0.02). 

The differences in milk yields among HG, LD, and HD groups by disease can be found in Table 7. Differences in daily milk production showed that HD cows would produce less milk than LD and HG cows. Daily milk loss per cow with a high SCC relative to healthy cows can be 1.07, 12.86, 11.23, 8.14, and 3.14 when diagnosed with metritis, mastitis, retained placenta, ketosis, and lameness. 

Similarly, HD versus LD milk data showed that daily decline in milk from cows with elevated SCC prior to dry-off, diagnosed with disease, to be 1.08, 11.36, 4.04, 6.18, and 1.74 for metritis, mastitis, retained placenta, ketosis and lameness, respectively. 

## 4. Discussion

We hypothesized that high SCC in the milk of dairy cow prior to drying off is linked to a higher incidence of postpartum disease, as well as changes in milk composition and milk yield. Cows with high SCC did, in fact, have a higher incidence of periparturient diseases, most notably ketosis. For all diseases studied, milk composition analysis revealed that cows with high SCC had a lower concentration of lactose prior to dry off. Concentrations of protein in the milk were also higher in the HD group prior to dry off, decreasing in the first week after parturition and even more in the second week of lactation. The number of somatic cells in the milk after parturition was higher in the HD group for cows with retained placenta. In mastitis cows, on the other hand, the LD group had higher SCC after parturition than the HD group. For diseases such as mastitis, retained placenta, and ketosis, milk yield was lower in the HD group.

### 4.1. Relation of SCC to Incidence of Periparturient Diseases

To the best of our knowledge, this is the first study to show an association between high SCC before drying off and the incidence of peripartum disease in dairy cows. The most intriguing finding from this study was that ketosis was more common in cows with high SCC before drying off. In fact, a cow with high SCC before drying off was 166% more likely than a cow with low SCC to develop ketosis in the first two weeks after parturition. High SCC in milk indicates subclinical mastitis. A possible explanation for cows with high SCC before drying off being more prone to ketosis or other periparturient diseases could be systemic endotoxemia during dry period. This hypothesis is based on the suggestion by Eckel and Ametaj [28] that there are three sources of bacterial endotoxins in dairy cows: mammary gland, reproductive tract, and rumen. In fact, the mammary gland is the only source of endotoxin during the dry period. The rumen and uterus are only sources of endotoxins after calving, when the uterus can become infected with pathogenic bacteria causing metritis immediately after calving, whereas grain feeding after parturition is associated with the release of large quantities of endotoxins in the rumen. We hypothesize that endotoxin is translocated from the mammary gland into the systemic circulation of cows with high SCC due to infection but also due to antibiotic treatment at drying off. This suggests that whereas the antibiotic kills the pathogenic bacteria in the mammary gland, the endotoxin released by the dead bacteria escapes into the systemic circulation and triggers a systemic inflammatory response. In fact, our laboratory [34] reported that cows with postpartum ketosis had higher serum BHBA concentrations 4 weeks before calving. We also showed that blood concentrations of tumor necrosis factor alpha (TNF-a), haptoglobin (Hp), and interleukin-6 (IL-6) were higher in pre-ketotic cows 4 weeks before calving and during disease diagnosis than in healthy controls at the start of ketosis (1–3 weeks after birth). Additionally, [35] confirmed those findings in ketotic cows, demonstrating increased blood concentrations of LPS prepartum and of serum amyloid A (SAA), Hp, and lipopolysaccharide binding protein (LBP) postpartum versus healthy controls.

Previous research on both natural and experimentally induced mastitis, in which LPS concentrations were observed in the plasma of dairy cows with mastitis, support the possibility of mammary gland endotoxin entering the systemic circulation [29,30]. A study from our laboratory [36] showed that 10 days before calving, blood levels of beta-hydroxybutyric acid (BHBA) increased after parenteral treatment with LPS at increasing doses around parturition. High levels of BHBA are known to be associated with ketosis in dairy cows [37]. In another study [34], we showed increases in serum BHBA in pre-ketotic cows 4 weeks before calving, suggesting that endotoxin insults could potentially contribute to increased BHBA. Beta-hydroxybutyric acid has been shown to inhibit NLRP3 macrophage inflammasome activation. When the NLRP3 inflammasome is activated, pro-inflammatory cytokines such as TNF-α, IL-1, IL-6 and acute phase proteins are released [38]. Consequently, the susceptibility to ketosis of cows with an elevated SCC could be attributed, inter alia, to the translocation of endotoxin into the systemic circulation before drying off and during the dry period, contributing to an increase in ketone bodies during the dry period to control the inflammatory response.

Odds ratio data showed that in cows with elevated SCC before drying off, the likelihoods of metritis, retained placenta, and lameness were increased by 43%, 56% and 31%, respectively. However, ***p***-values were not statistically significant, implying that other factors may contribute to the incidence of postpartum disease. For example, abortion, twins, dystocia, and short gestation have been associated with retained placenta. Because some of the cows in this study suffered from multiple periparturient diseases, it makes sense that cows with retained placenta are also more prone to metritis. The occurrence of metritis can also be influenced by bacterial infection of the uterus after calving [23]. Postpartum grain feeding has been strongly associated with an increased incidence of lameness [39]. Elevated SCC prior to drying off (i.e., subclinical mastitis) may play a role in the etiopathology of metritis, retained placenta, and lameness, possibly through translocation of bacterial endotoxins from mammary glands with elevated SCC; however, more research is needed on this topic.

Bacterial endotoxins and inflammatory cytokines, released from the mammary gland of cows with elevated SCC into the systemic circulation, may contribute or increase the likelihood of these three periparturient diseases. Systemic and local administrations of LPS resulted in lesions in the corium and epidermis of the hoof region, implicating a role of endotoxin in the pathogenesis of lameness [24]. In addition, intermittent and increasing doses of LPS over 3 weeks before calving is associated with an increased risk of retained placenta [36]. Exposure of neutrophils to LPS can induce LPS tolerance, leading to decreased expression of TLR4 and compromised host immune response [40]. Tolerance to LPS may explain why cows with high SCC were 56% more likely to retain the placenta.

The reproductive tract, along with the mammary gland and rumen of dairy cows, has been suggested as a source of endotoxin by [28]. Conversely, mammary gland infections can affect the reproductive tract in dairy cows. For example, induction of mastitis in cows with *Escherichia coli* (*E. coli*) LPS, has been shown to decrease follicular estrogen, androstenedione, and progesterone by 40%, 13%, and 35%, respectively [41]. The latter authors also found a 56% reduction in circulating concentrations of estrogen in mastitis induced by *Staphylococcus aureus* (*S. aureus*). Furthermore, systemic endotoxin can impair the release of hormones from the hypothalamus and pituitary gland. Battagila et al. [42] demonstrated this in ewes by slowing or blocking luteinizing hormone and follicular-stimulating hormone surges, which interfere with the pre-ovulatory increase in estrogen. Similarly, in dairy cows, this type of delayed response can lead to poor reproductive performance. Therefore, cows with high SCC and increased risk of metritis may also have poor reproductive performance.

Additionally, previous research by our team found changes in innate immune reactants from 8 and 4 weeks prior to parturition in cows with retained placenta, metritis, lameness, ketosis, and mastitis [10,26,34,43,44]. Results from previous studies indicate that dairy cows prior to calving are in a low-grade chronic inflammatory state starting as early as 2 months prior to parturition. Although we did not analyze changes in innate immune reactants prior to cows drying off, it is hypothesized that cows with elevated SCC experience an inflammatory state at the end of the lactation cycle and during the dry period. The odds ratios for the incidence of mastitis for the LD and HD groups were 0.98 and 1.02, respectively, suggesting other factors may predispose cows to post-partum mastitis. These could include the inefficiency of the dry cow therapeutic treatment, insufficient sealant secretion in the teat canal, or sealant before depletion prior to calving [19].

### 4.2. Alterations in Milk Composition

Cows who had high SCC before being diagnosed with post-partum disease had significantly higher SCC in their milk postpartum. Significant differences in SCC between groups and diseases were expected. The HD group that was diagnosed with retained placenta after parturition had elevated SCC after calving. In comparison, the SCC after parturition in the LD group remained <200,000 cells/mL. Previously, elevated SCC were observed in cows diagnosed with retained placenta during the diagnosis week, however, the SCC were within the normal range [10]. The cows in the current study had a significantly higher SCC for the HD group at 1 week after calving, which was the week of diagnosis of retained placenta. At 2 weeks postpartum, the SCC was slightly higher than the subclinical mastitis cut off value. The possible reason for the high SCC in all three time periods measured in the retained placenta group could be attributed to the number of diseases the cows were experiencing when diagnosed with retained placenta. The effect of the number of diseases (multiple diseases) was taken into account when modeling SCC for retained placenta, but this effect was not significant, suggesting that other factors may contribute to the increase in SCC after parturition in cows with retained placenta.

The number of somatic cells in the mastitis group increased after parturition in the LD and HD groups. The LD group may have had a new IMI during the dry off period, leading to increased SCC in milk after parturition. According to [19], 95% of all new IMI occur between 2 and 3 weeks before calving. The incidence of IMI has been reported to be highest at the start of the dry off period and towards the end of the pregnancy [45]. Furthermore, the type of bacterial strain could be a factor in low SCC cows becoming sick or high SCC cows still having a high incidence of mastitis after drying off. For example, bacterial infection by *S. aureus* has been found to be more frequent at the start of lactation [46]. Moreover, the host immune response to *S. aureus* infections has been reported to be weaker (Bannerman et al., 2004). The slow response could be attributed to *S. aureus* biofilm formation, which protects the pathogen from neutrophil phagocytosis [47]. It would be interesting to perform an analysis of the bacterial strains of the milk microbiota in cows with low and high SCC in order to determine the type of pathogens that can cause mastitis.

Cows in the HD group prepartum who were diagnosed with lameness, ketosis, or metritis after parturition had normal SCC in the milk postpartum. It has previously been shown that cows diagnosed with metritis, ketosis, and lameness have a higher SCC during disease diagnosis than healthy cows; however, the number of SCC was within the normal range, below 200,000 cells/mL, which is consistent with the current study [34,43,44]. Several authors have suggested that the decrease in milk SCC for the HD group may be due to the sensation of pain associated with hoof inflammation, and that cows may be standing more than lying down, as the lying position is extremely painful for lame cows [48]. However, this remains controversial. Archer et al. [25] attempted to find an association between milk SCC and lameness. Those authors showed that lame cows had lower SCC than non-lame cows and concluded that lame cows spend more time standing than lying down, reducing mammary gland exposure to bedding bacteria [25]. 

In all diseases, lowered lactose concentrations in the milk were observed in the HD group. The mammary gland consists of a network of alveoli that are lined with mammary epithelial cells (MECs) that secrete milk and are connected by tight junctions to prevent milk from entering into the systemic circulation [49]. During infections, an influx of leukocytes into the mammary gland to remove pathogens causes an increase in the gap between the epithelial cells [15,50]. The wider gap allows lactose to escape into the systemic circulation, which is supported by several authors who have observed increases in lactose concentrations in the blood and urine of cows with mastitis [15,50]. Other factors that may contribute to the degradation of lactose in milk include the ability of some bacterial serotypes to use lactose for their needs [15], as well as physical damage to the MECs, resulting in reduced lactose synthesis. Furthermore, lactose acts as the primary osmotic regulator for milk synthesis [8], so if lactose concentrations in milk decrease, milk production will also decrease in cows with a high SCC. In addition, pro-inflammatory cytokines, as well as pathogens and their associated endotoxins, are believed to play a role in lactose synthesis. TNF-a, a potent pro-inflammatory cytokine, has been shown to influence the lactose secretion pathway by downregulating lactose synthesis-related genes such as a-lactalbumin gene and the degradation glucose transporter-1 (GLUT1) from the basolateral membrane [51]. As a result, the decrease in lactose in cows with high SCC can be attributed to an increase in inflammatory mediators during infection, as well as the suppression of genes involved in lactose synthesis. 

Moreover, we identified changes in milk protein concentrations for lameness over all time periods, ketosis in the first and second weeks after calving, and retained placenta that had a tendency prior to dry off period. Interestingly, for all diseases studied, concentrations of protein in the milk, in the HD group were higher before dry-off and lowered after calving. During a mammary gland infection, the influx of blood-borne proteins, such as serum albumin and immunoglobulins, increases during a mammary gland infection, due to the immune response [7,8,15]. A study using proteomics in bovine milk from mastitis cows, identified proteins involved in the immune response, including lactoferrin, transferrin, fibrinogen, apolipoprotein A1, glycosylation-dependent cell adhesion molecule-1, peptidoglycan recognition proteins, as well as cathelicidin-1 [52]. Furthermore, lactoferrin (an iron-binding protein) has been reported to increase nearly 100-fold during the involution phase of the dry period to prevent iron utilization by iron-consuming bacteria. Lameness has previously been shown to be associated with a decrease in both milk protein and milk fat.

Additionally, key milk proteins have been shown to decrease during infection, including casein and whey proteins such as a-lactalbumin and b-lactoglobulin. The decrease in milk proteins can be caused by bacterial proteinases, leakage of proteins from the mammary gland, and a decrease in synthesis due to damage to the MECs [8]. Furthermore, secretion of TNF-a in rats has been shown to suppress both transcriptional and posttranscriptional gene expression for b-casein [53]. Under normal conditions, TNF-a is important for the proliferation and differentiation of MECs in the mammary glands of rats [54]. In dairy cows, the increase in TNF-a along with other pro-inflammatory cytokines is important for the host’s immune response to infection and can therefore inhibit the expression of milk proteins.

Strong associations between milk protein concentration and energy balance have been shown, with low milk protein content reflecting negative energy balance (NEB) and poor reproductive performance [55]. A negative energy balance is strongly associated with ketosis, especially during early lactation when the energy demand for milk production is high [56]. We observed a reduction in milk protein after calving in cows diagnosed with ketosis, with the HD group showing the lowest concentration. The decrease in milk protein suggests that cows suffer NEB and have insufficient feed intake. Additionally, neutrophil granules contain both enzymes and antibacterial peptides which are important for killing bacteria during infection but can also change milk protein synthesis during infection [57].

Additionally, endotoxins or pro-inflammatory cytokines translocated from the mammary gland of cows with high SCC cows during the dry period may play a role in NEB and indirectly contribute to low milk protein, leading to the development of ketosis. Systemic circulation of pro-inflammatory cytokines triggers the expression of acute phase proteins from the liver [58]. Reports from LPS-induced mastitis models have shown induction of transcriptome response by the liver and increased expression of acute phase protein-related genes [59]. Zhang et al., (2016) observed an upregulation of TNF-a and serum amyloid A in ketotic cows during the week of disease diagnosis and 8 and 4 weeks before calving. The current study has confirmed that the onset of ketosis is significantly associated with high SCC before drying off, and decreased milk protein concentration in the HD group, further supporting this hypothesis. 

In the present study, there was a difference in the FPR in the HD group diagnosed with ketosis 2 weeks after calving. The FPR has been proposed as an indicator for the diagnosis of cows in ketosis [60]. Several authors have proposed different threshold values to diagnose ketosis using FPR values. Heuer et al. [60] found that cows with an FPR of >1.5 had an increased risk of clinical ketosis. The same authors also reported that at that cut-off there was a higher incidence of other post-partum diseases, including displaced abomasum, ovarian cysts, lameness, and mastitis [60]. Other researchers have reported an increased incidence of retained placenta, displaced abomasum, metritis, endometritis, and risk of culling at an FPR > 2.0 at 7 DIM [61]. Cows in the HD group diagnosed with ketosis in our study had higher FPR before drying off (1.33 ± 0.19), 1 week (1.57 ± 0.20) and at 2 weeks (1.71 ± 0.19) after parturition compared to cows from the HG and LD groups, further showing that cows with high SCC before drying off are more prone to onset of ketosis.

Changes in milk fat content showed no differences between the groups for four diseases, whereas cows with metritis tended to have higher milk fat content before drying off. Milk fat concentrations were higher in the HD group of pre-metritic cows. Previously, milk fat concentrations were lowest in cows during the week of metritis diagnosis [44]. On the other hand, [50] reported an increase in milk fat and a decrease in milk lactose during mammary gland infections. Enlarged gaps between the MEC tight junctions lead to leakage of milk components from the mammary gland; however, because milk fat globules are too large to move through the tight junctions, they remain in the mammary gland [14]. The increase in milk fat concentration of HD group cows before drying off can be attributed to the decrease in other milk components during infection and is less likely to be associated with the occurrence of metritis.

There was no difference in MUN and TS concentrations between the three disease incidence groups (HG, HD, and LD) and will not be discussed any further here.

### 4.3. Alterations in Milk Production

Milk production was shown to be lower in all diseases studied during the first 60 DIM, with retained placenta, mastitis, and ketosis being the most important in the loss of production in cows with a high SCC at drying off. We have previously found that cows with metritis, mastitis, retained placenta, lameness, and ketosis have lower daily milk production [10,26,34,43,44]. The emergence of new IMI can have a significant impact on both milk synthesis and secretion, leading to a decrease in milk yield [62]. Decreased milk production associated with elevated SCC may be due to bacterial infection, causing influx of leukocytes in the mammary gland and the secretion of inflammatory mediators, leading to rupture of tight junctions [15,50]. 

Furthermore, decreased lactose synthesis may contribute to the loss of milk production [8], which was identified in the HD group of this study. Further to that, prolactin released from anterior pituitary gland regulates both cellular and humoral immune responses as well as milk yield. There is an increase in prolactin secretion towards the end of pregnancy, which stimulates the proliferation of the alveoli in the mammary gland, resulting in increased milk production after calving [16]. External LPS has been shown to activate the hypothalamic-pituitary–adrenocortical axis and cause the release of pro-inflammatory cytokines [63]. Additionally, pro-inflammatory cytokines have been shown to inhibit prolactin secretion in rodents [64]. Therefore, it is hypothesized that high SCC in cows with lower postpartum milk production may be related to suppression of prolactin secretion by the pituitary. 

The dry period is critical for the regeneration of milk-secreting cells in the mammary gland [16]. Our results on reduced milk production in cows with high SCC suggest that inflammation of the mammary gland before drying off influences milk production and milk composition in the following lactation. In addition, cows with a high SCC had lower daily milk production than cows with low SCC. This is particularly important for producers, as cows with a high SCC before dry period can lead to large economic losses during the next lactation. The additional management and labor costs, the higher culling rate, and milk waste are all negative effects of the high SCC on the farm profitability [65]. As a result, better management of late lactating dairy cows may be needed before the dry off period to increase future production and reduce disease incidence. This includes screening the cows for SCC prior to drying them off. Finally, further research on the etiological factors of cows with high SCC and their association with the incidence of periparturient diseases is needed to gain a better understanding of the pathological mechanisms involved in the disease process, the health of dairy cows during the periparturient period, and reduce to a minimum the loss of production. 

## 5. Conclusions

The results of this study indicate that dairy cows with elevated SCC prior to drying off were highly susceptible to postpartum ketosis. Low protein concentrations in postpartum milk were significantly associated with high SCC in cows diagnosed with ketosis, possibly due to NEB status. In addition, significant differences were found in the FPR for the HD group at 2 weeks postpartum in cows that were diagnosed with ketosis. Although not significant, the incidence of metritis, retained placenta, and lameness were more likely to occur in cows with high SCC before drying off; however other factors can also contribute to the incidence of disease. 

Milk composition was shown to be altered in high SCC cows where lactose was lower at all sampling time points, and protein concentrations were higher prior to dry of and lower after parturition. Somatic cell counts were significantly greater for high SCC cows prior to dry off for all diseases. After parturition, SCC were greater for the HD group with retained placenta, which could be related to other factors. Somatic cell counts after parturition for ketosis, metritis, and lameness groups were within normal ranges (<200,000 cells/mL), while SCC were not significant between LD and HD groups diagnosed with mastitis. 

Milk production after parturition was also found to be significantly lower for cows with high SCC prior to dry off that were diagnosed with mastitis, ketosis, and retained placenta. Although not statistically significant, milk production for high SCC cows was numerically lower for metritis and lameness, indicating milk yield potentially could be affected after parturition if cows are dried off with high SCC. 

## Figures and Tables

**Table 1:** Frequencies of the incidence rate for cows diagnosed with periparturient disease between SCC groups and number of diseases.

	SCC Group *			Number of Diseases ^4,^*	
Disease	Low ^1^	High ^2^	Total ^3^	Single	Multiple
Metritis	38 (27.14%)	21 (15.00%)	59 (42.14)	17 (12.14)	42 (30.00)
Retained Placenta	12 (8.57%)	8 (5.71%)	20 (14.29)	3 (2.14)	17 (12.14)
Mastitis	28 (20.00%)	13 (9.29%)	41(29.29)	11 (7.86)	30 (21.43)
Ketosis	24 (17.14%)	21 (15.00%)	45 (32.14)	18 (12.86)	27 (19.29)
Lameness	15 (10.71%)	7 (5.00%)	22 (15.71)	3 (2.14)	19 (13.57)
Healthy	24 (17.14%)	7 (5.00%)	31 (22.14)	-	-

^1^ Low: SCC prior to dry off <200,000 cells/mL, percentage of total cows diagnosed with disease; ^2^ High: SCC prior to dry off >200,000 cells/mL, percentage of total cows diagnosed with disease; ^3^ Total cows diagnosed with disease and percentage of total population, ^4^ Number of diseases: cows diagnosed with 1 disease (single) and >2 diseases (multiple), exception are healthy cows which had no incidence of disease. * Percentages (brackets) are calculated out of the total population (n = 140). Note that cows were diagnosed with multiple diseases, and there is overlap within other disease groups, except for SCC groupings and single disease (i.e., The same cow diagnosed with retained placenta and metritis is considered multiple). Therefore, sum of the percentages will not equal 100.

**Table 2:** Comparisons of odd ratios between low SCC and high SCC cows that were diagnosed with post-partum disease.

	Odds Ratio				
Disease	Low ^1^	95% CI ^2^	High ^3^	95% CI	*p*-Value
Metritis	0.70	0.34–1.44	1.43	0.69–2.96	0.33
Retained Placenta	0.64	0.24–1.71	1.56	0.59–4.13	0.38
Mastitis	0.98	0.45–2.15	1.02	0.47–2.23	0.96
Ketosis	0.38	0.18–0.80	2.66	1.26–5.65	0.01
Lameness	0.96	0.36–2.57	1.31	0.39–2.76	0.94

^1^ Low: SCC prior to dry off <200,000 cells/mL; ^2^ 95% CI: 95% Wald confidence limits. ^3^ High: SCC prior to dry off >200,000 cells/mL.

**Table 3:** Alterations in milk composition (fat, protein, fat:protein ratio, milk urea nitrogen (MUN), total solids (TS), and somatic cell counts (SCC) one week prior to dry off in dairy cows diagnosed with postpartum disease.

Disease	Health	Milk Composition Prior to Dry off						
	Status	SCC 10^3^ Cells/mL	Fat %	Protein %	FPR	Lactose %	MUN mg/dL	TS %
Metritis ^4^	HG ^1^	66.40 ± 17.07 ^a^	4.13 ± 0.56 ^a^	3.49 ± 0.09 ^b^	1.19 ± 0.1	4.46 ± 0.09 ^a^	12.71 ± 1.06	13.08 ± 0.58
	LD ^2^	15.10 ± 0.85 ^a^	4.79 ± 0.82 ^a,b^	3.75 ± 0.12 ^a^	1.26 ± 0.22	4.47 ± 0.13 ^a^	12.23 ± 1.43	14.06 ± 0.82
	HD ^3^	509.00 ± 64.70 ^b^	6.52 ± 0.96 ^b^	3.75 ± 0.12 ^a^	1.73 ± 0.26	4.01 ± 0.12 ^b^	14.39 ± 1.55	15.10 ± 0.85
	*p*-value	<0.01	0.09	0.11	0.17	0.01	0.55	0.15
Retained	HG ^1^	66.40 ± 28.99 ^a^	4.13 ± 0.36	3.40 ± 0.11 ^a^	1.19 ± 0.11	4.46 ± 0.09 ^a^	13.06 ± 0.99	13.08 ± 0.41
Placenta ^5^	LD ^2^	47.29 ± 35.82 ^a^	4.59 ± 0.55	3.73 ± 0.14 ^a^	1.23 ± 0.16	4.47 ± 0.13 ^a^	14.49 ± 1.34	13.86 ± 0.63
	HD ^3^	851.86 ± 152.03 ^b^	4.77 ± 0.56	3.91 ± 0.18 ^b^	1.26 ± 0.16	4.06 ± 0.13 ^b^	13.24 ± 1.49	13.67 ± 0.62
	*p*-value	<0.01	0.57	0.06	0.93	0.05	0.65	0.52
Mastitis ^6^	HG ^1^	66.40 ± 50.03 ^a^	4.13 ± 0.37	3.49 ± 0.07 ^a^	1.19 ± 0.11	4.46 ± 0.09 ^a^	12.71 ± 1.14	13.08 ± 0.41
	LD ^2^	74.63 ± 72.62 ^a^	3.89 ± 0.50	3.61 ± 0.10 ^a^	1.07 ± 0.15	4.61 ± 0.13 ^a^	13.10 ± 1.59	13.09 ± 0.56
	HD ^3^	1554.50 ± 331.44	4.28 ± 0.52	3.69 ± 0.10 ^b^	1.16 ± 0.15	4.07 ± 0.12 ^b^	12.46 ± 1.55	13.09 ± 0.56
	*p*-value	<0.01	0.86	0.24	0.82	0.01	0.96	1.0
Lameness ^7^	HG ^1^	67.79 ± 10.51 ^a^	4.25 ± 0.42	3.41 ± 0.10 ^a^	1.19 ± 0.12	4.37 ± 0.10 ^a^	13.03 ± 1.05	13.21 ± 0.51
	LD ^2^	65.00 ± 14.56 ^a^	3.78 ± 0.56	3.79 ± 0.14 ^b^	1.03 ± 0.16	4.54 ± 0.14 ^a^	14.05 ± 1.54	12.90 ± 0.71
	HD ^3^	351.29 ± 33.86	4.62 ± 0.62	4.10 ± 0.15 ^c^	1.15 ± 0.17	3.98 ± 0.13 ^b^	13.23 ± 1.50	13.55 ± 0.73
	*p*-value	<0.01	0.61	<0.01	0.73	0.01	0.85	0.82
Ketosis ^8^	HG ^1^	66.40 ± 52.32 ^a^	4.13 ± 0.39	3.49 ± 0.08	1.19 ± 0.12	4.46 ± 0.11 ^a^	12.71 ± 1.03	13.08 ± 0.46
	LD ^2^	98.13 ± 87.09 ^a^	3.98 ± 0.53	3.55 ± 0.11	1.12 ± 0.16	4.43 ± 0.16 ^a^	11.07 ± 1.32	12.98 ± 0.62
	HD ^3^	1292.71 ± 337.92	4.87 ± 0.63	3.64 ± 0.12	1.33 ± 0.19	3.73 ± 0.1 ^b^	10.82 ± 1.39	13.2 ± 0.67
	*p*-value	<0.01	0.49	0.55	0.68	<0.01	0.47	0.96

^1^ Healthy group (n = 15): cows that were low SCC and had no incidence of disease throughout study period; ^2^ Low-disease: SCC < 200,000 cells/mL prior to dry off, ^3^ High-disease: SCC > 200,000 cells/mL prior to dry off, ^4^ Metritis: LD (n = 8), HD (n = 8); ^5^ Retained placenta: LD (n = 7), HD (n = 7); ^6^ Ketosis: LD (n = 8), HD (n = 7); ^7^ Lameness: LD (n = 7), HD (n = 7); ^8^ Mastitis: LD (n = 8), HD (n = 8); ^a–c^ Numbers with different superscripts with difference *p* < 0.05.

**Table 4:** Alterations in milk composition (fat, protein, fat:protein ratio, milk urea nitrogen (MUN), total solids (TS), and somatic cell counts (SCC)) at 1 week after parturition in dairy cows diagnosed with post-partum disease.

Disease	Health	Milk Composition at +1 Weeks						
	Status	SCC 10^3^ Cells/mL	Fat %	Protein %	FPR	Lactose %	MUN mg/dL	TS %
Metritis ^4^	HG ^1^	54.07 ±10.80	4.56 ± 0.57	3.67 ± 0.07	1.25 ± 0.16	4.43 ± 0.05 ^a^	13.11 ± 0.61	13.86 ± 0.58
	LD ^2^	51.38 ± 14.42	4.50 ± 0.77	3.84 ± 0.10	1.19 ± 0.21	4.42 ± 0.06 ^a^	11.26 ± 0.77	13.96 ± 0.80
	HD ^3^	53.00 ± 14.65	5.49 ± 0.86	3.55 ± 0.10	1.57 ± 0.25	4.19 ± 0.06 ^b^	12.66 ± 0.82	14.74 ± 0.80
	*p*-value	0.99	0.60	0.12	0.41	0.01	0.20	0.66
Retained	HG ^1^	54.07 ± 78.73 ^a^	4.56 ± 0.49	3.81 ± 0.24	1.25 ± 0.15	4.44 ± 0.12 ^a^	14.09 ± 1.27	13.86 ± 0.50
Placenta ^5^	LD ^2^	41.57 ± 101.06 ^a,b^	4.80 ± 0.73	3.73 ± 0.28	1.29 ± 0.22	4.31 ± 0.18 ^a,b^	14.72 ± 1.53	13.96 ± 0.74
	HD ^3^	1359.86 ± 578.00 ^b^	5.68 ± 0.79	3.78 ± 0.34	1.60 ± 0.24	3.96 ± 0.17 ^a^	12.28 ± 1.59	14.91 ± 0.76
	*p*-value	0.06	0.46	0.97	0.42	0.10	0.57	0.49
Mastitis ^6^	HG ^1^	54.07 ± 96.00	4.56 ± 0.49	3.67 ± 0.17	1.25 ± 0.13	4.44 ± 0.05 ^a^	13.11 ± 0.75	13.86 ± 0.48
	LD ^2^	1993.63 ± 798.24	4.54 ± 0.66	3.68 ± 0.23	1.24 ± 0.18	4.40 ± 0.07 ^a^	12.02 ± 1.00	13.87 ± 0.67
	HD ^3^	1396.00 ± 667.96	4.94 ± 0.69	4.04 ± 0.24	1.32 ± 0.19	4.15 ± 0.06 ^b^	12.15 ± 1.00	14.37 ± 0.67
	*p*-value	0.15	0.89	0.42	0.94	<0.01	0.61	0.80
Lameness ^7^	HG ^1^	47.86 ± 9.63	4.50 ± 0.42	3.70 ± 0.10 ^a^	1.25 ± 0.12	4.37 ± 0.05	13.46 ± 0.79	13.91 ± 0.48
	LD ^2^	62.57 ± 15.58	4.89 ± 0.72	3.87 ± 0.13 ^a^	1.04 ± 0.17	4.45 ± 0.06	12.03 ± 1.06	13.55 ± 0.67
	HD ^3^	69.29 ± 16.39	4.89 ± 0.67	3.29 ± 0.12 ^b^	1.28 ± 0.96	4.38 ± 0.06	13.19 ± 1.10	13.12 ± 0.66
	*p*-value	0.47	0.85	<0.01	0.57	0.63	0.57	0.63
Ketosis ^8^	HG ^1^	54.07 ± 12.39	4.65 ± 0.48	3.67 ± 0.08 ^a^	1.25 ± 0.12	4.44 ± 0.04	13.11 ± 0.79	13.86 ± 0.50
	LD ^2^	61.38 ± 18.08	4.03 ± 0.63	3.48 ± 0.10 ^a^	1.11 ± 0.16	4.43 ± 0.05	12.53 ± 1.06	13.03 ± 0.66
	HD ^3^	87.00 ± 23.01	4.28 ± 0.65	3.26 ± 0.11 ^b^	1.57 ± 0.20	4.33 ± 0.06	13.47 ± 1.17	14.99 ± 0.73
	*p*-value	0.40	0.73	0.01	0.20	0.25	0.83	0.55

^1^ Healthy group (n = 15): cows that were low SCC and had no incidence of disease throughout study period; ^2^ Low-disease: SCC < 200,000 cells/mL prior to dry off; ^3^ High-disease: SCC > 200,000 cells/mL prior to dry off; ^4^ Metritis: LD (n = 8), HD (n = 8); ^5^ Retained placenta: LD (n = 7), HD (n = 7); ^6^ Ketosis: LD (n = 8), HD (n = 7); ^7^ Lameness: LD (n = 7), HD (n = 7); ^8^ Mastitis: LD (n = 8), HD (n = 8); ^a,b^ Numbers with different superscripts with difference *p* < 0.05.

**Table 5:** Alterations in milk composition (fat, protein, fat:protein ratio, milk urea nitrogen (MUN), total solids (TS), and somatic cell counts (SCC)) at 2 weeks after parturition in dairy cows diagnosed with post-partum disease.

Disease	Health	Milk Composition at +2 Weeks						
	Status	SCC 10^3^ Cells/mL	Fat %	Protein %	FPR	Lactose %	MUN mg/dL	TS %
Metritis ^4^	HG ^1^	35.53 ± 8.49	3.38 ± 0.22	3.32 ± 0.06	1.02 ± 0.07 ^a^	4.58 ± 0.03 ^a^	12.67 ± 0.78	12.34 ± 0.23
	LD ^2^	22.63 ± 9.27	3.31 ± 0.30	3.36 ± 0.08	0.99 ± 0.09 ^a^	4.66 ± 0.05 ^a^	12.06 ± 1.04	12.37 ± 0.31
	HD ^3^	30.13 ± 10.70	3.94 ± 0.32	3.16 ± 0.08	1.25 ± 0.10 ^b^	4.45 ± 0.04 ^b^	13.12 ± 1.09	12.64 ± 0.32
	*p*-value	0.64	0.26	0.17	0.10	0.01	0.78	0.73
Retained	HG ^1^	37.58 ± 22.99 ^a^	3.44 ± 0.41	3.46 ± 0.12	1.02 ± 0.08	4.55 ± 0.09	12.77 ± 1.10	12.38 ± 0.49
Placenta ^5^	LD ^2^	43.57 ± 32.41 ^a^	3.57 ± 0.55	3.37 ± 0.14	1.07 ± 0.13	4.50 ± 0.11	11.06 ± 1.12	12.45 ± 0.64
	HD ^3^	223.86 ± 73.47 ^b^	4.56 ± 0.62	3.09 ± 0.15	1.34 ± 0.14	4.36 ± 0.11	11.89 ± 1.44	13.34 ± 0.66
	*p*-value	0.02	0.28	0.27	0.15	0.42	0.53	0.48
Mastitis ^6^	HG ^1^	37.58 ± 67.26 ^b^	3.44 ± 0.35	3.33 ± 0.09	1.02 ± 0.10	4.55 ± 0.10	13.21 ± 1.00	12.38 ± 0.36
	LD ^2^	939.37 ± 411.81 ^a^	3.50 ± 0.43	3.31 ± 0.10	1.07 ± 0.14	4.60 ± 0.13	12.03 ± 1.17	12.55 ± 0.45
	HD ^3^	666.50 ± 346.88 ^a^	4.04 ± 0.46	3.30 ± 0.10	1.23 ± 0.15	4.32 ± 0.12	10.23 ± 1.08	12.77 ± 0.45
	*p*-value	0.23	0.54	0.96	0.47	0.25	0.16	0.80
Lameness ^7^	HG ^1^	34.29 ± 15.58	3.35 ± 0.26	3.38 ± 0.09 ^a^	1.02 ± 0.08	4.57 ± 0.03	12.66 ± 0.88	12.28 ± 0.28
	LD ^2^	91.29 ± 35.95	3.68 ± 0.45	3.39 ± 0.11 ^a^	0.98 ± 0.12	4.52 ± 0.05	11.80 ± 1.20	12.37 ± 0.40
	HD ^3^	58.71 ± 28.83	4.18 ± 0.42	2.93 ± 0.10 ^b^	1.31 ± 0.14	4.45 ± 0.05	12.96 ± 1.26	12.45 ± 0.40
	*p*-value	0.28	0.27	<0.01	0.13	0.16	0.78	0.94
Ketosis ^8^	HG ^1^	35.53 ± 9.04	3.33 ± 0.28	3.32 ± 0.07 ^a^	1.02 ± 0.10 ^a^	4.58 ± 0.04 ^a^	12.67 ± 0.79	12.34 ± 0.28
	LD ^2^	41.50 ± 13.38	3.31 ± 0.39	3.12 ± 0.10 ^b^	1.21 ± 0.15 ^a^	4.55 ± 0.05 ^a^	11.16 ± 1.01	12.49 ± 0.39
	HD ^3^	44.43 ± 14.80	3.91 ± 0.42	2.80 ± 0.10 ^c^	1.71 ± 0.19 ^b^	4.42 ± 0.05 ^b^	11.96 ± 1.12	13.11 ± 0.43
	*p*-value	0.85	0.46	<0.01	<0.01	0.09	0.52	0.32

^1^ Healthy group (n = 15): cows that were low SCC and had no incidence of disease throughout study period; ^2^ Low-disease: SCC < 200,000 cells/mL prior to dry off; ^3^ High-disease: SCC > 200,000 cells/mL prior to dry off, ^4^ Metritis: LD (n = 8), HD (n = 8); ^5^ Retained placenta: LD (n = 7), HD (n = 7); ^6^ Ketosis: LD (n = 8), HD (n = 7); ^7^ Lameness: LD (n = 7), HD (n = 7); ^8^ Mastitis: LD (n = 8), HD (n = 8); ^a–c^ Numbers with different superscripts with difference *p* < 0.05.

**Table 6:** Total milk yields comparison for 60 days in milk (DIM) among diseases and among healthy, low-disease, and high-disease groups.

	Mean Total Yield for 60 Days in Milk (DIM, kg)			*p*-Value		
Disease	HG ^1^	LD ^2^	HD ^3^	HS ^4^	PY ^5^	HS × PY ^6^
Metritis	2748.21± 68.90	2748.07 ± 89.65	2683.27 ± 93.00	0.84	0.02	NS ^7^
Retained placenta	2716.81 ± 153.14	2285.54 ± 216.57	2042.99 ± 216.57	0.04	NS	NS
Mastitis	2742.51 ± 122.88	2652.61 ± 158.63	1970.88 ± 177.47	<0.01	<0.01	0.01
Lameness	2716.81± 86.85	2633.04 ± 122.83	2528.36 ± 122.83	0.46	NS	NS
Ketosis	2789.97 ± 107.43	2672.33 ± 139.34	2301.81 ± 152.20	0.05	<0.01	NS

^1^ Healthy group (n = 15): cows that were low SCC and had no incidence of disease throughout study period; ^2^ Low-disease: SCC < 200,000 cells/mL prior to dry off; ^3^ High-disease: SCC > 200,000 cells/mL prior to dry off; ^4^ HS = effect of health status; ^5^ PY = effect of previous yield for 305 DIM; ^6^ HS × PY = effect of health status and previous yield; ^7^ NS = no significance; variable showed no significance in the statistical model and was removed.

**Table 7:** Milk yield differences between groups for each disease for daily yield and total yield for 60 DIM per cow and for 100 cows.

		Disease				
	Group	Metritis	Mastitis	Retained Placenta	Ketosis	Lameness
Difference in total milk yield for 60 DIM per cow (kg/60 d) ^4^	LD ^1^ vs. HG ^2^	−0.14	−89.90	−431.27	−117.64	−83.77
	HD ^3^ vs. HG	−64.49	−771.63	−673.82	−488.16	−188.45
	HD vs. LD	−64.80	−681.73	−242.55	−370.52	−104.68
Difference in daily milk yield per cow (kg/d) ^5^	LD vs. HG	−0.0023	−1.50	−7.19	−1.96	−1.40
	HD vs. HG	−1.07	−12.86	−11.23	−8.14	−3.14
	HD vs. LD	−1.08	−11.36	−4.04	−6.18	−1.74
Difference in daily milk yield per 100 cows (kg/d) ^6^	LD vs. HG	−0.23	−150	−719.00	−196	−140
	HD vs. HG	−107	−1286	−1123	−814	−314
	HD vs. LD	−108	−1136	−404	−618	−174
Difference in total milk yield for 60 DIM per 100 cows (kg/60 d) ^7^	LD vs. HG	−13.80	−9000	−43,140	−11,760	−8400
	HD vs. HG	−6420	−77,160	−67,380	−48,840	−18,840
	HD vs. LD	−6480	−68,160	−24,240	−37,080	−10,440

^1^ Low-disease: SCC < 200,000 cells/mL prior to dry off; ^2^ High-disease: SCC > 200,000 cells/mL prior to dry off; ^3^ Healthy group (n = 15): cows that were low SCC and had no incidence of disease throughout study period; ^4^ Subtraction of total yield from Table 2, Table 3, Table 4, Table 5 and Table 6 for LD and HD, respectively from HG group to get difference in total milk yield for 60 DIM per cow; Negative values indicate how much less LD and HD are producing compared to healthy, and how much less HD are producing compared to LD; ^5^ Values calculated by dividing difference in total yield by 60 days in milk (DIM); ^6^ Values calculated by multiplying daily yield by 100 to determine loss per day per 100 cows; ^7^ Values calculated by multiplying daily yield per 100 cows by 60 to give total milk losses for 60 DIM per 100 cows.

## Data Availability

All data generated are presented in this article.

## Supplementary Materials

The following supporting information can be downloaded at: https://www.mdpi.com/article/10.3390/vetsci9110624/s1, Supplementary Table S1: Frequency of cows diagnosed with disease for parity; Supplementary Figure S1: Comparisons of somatic cell counts (10^3^ cells/mL) between healthy group (HG, blue), low disease (LD, orange), and high disease (HD, grey) for cows diagnosed with metritis, retained placenta, ketosis, lameness, and mastitis at (A) Prior to dry off (~1 week before date of dry off); (B) 1 week after parturition; (C) 2 weeks after parturition; Supplementary Figure S2: Comparisons of lactose concentrations (%) between healthy group (HG, blue bar), low disease (LD, orange bar), and high disease (HD, grey bar) for cows diagnosed with metritis, retained placenta, ketosis, lameness, and mastitis at (A) Prior to dry off (~1 week before date of dry off); (B) 1 weeks after parturition; (C) 2 weeks after parturition; Supplementary Figure S3: Comparisons of protein concentrations (%) between healthy group (HG, blue bar), low disease (LD, orange bar), and high disease (HD, grey bar) for cows diagnosed with metritis, retained placenta, ketosis, lameness, and mastitis at (A) Prior to dry off (~1 week before date of dry off); (B) 1 weeks after parturition; (C) 2 weeks after parturition; Supplementary Figure S4: Concentrations of somatic cell counts with (A) metritis, (B) retained placenta, (C) ketosis, (D) lameness, and (E) mastitis between healthy, low-disease, and high-disease (LSM ± SEM; HS = effect of health status; Wk = effect of week; Wk × HS = week × health status interaction); Supplementary Figure S5: Concentrations of lactose in the milk of cows with (A) metritis, (B) retained placenta, (C) ketosis, (D) lameness, and (E) mastitis between healthy, low-disease, and high-disease (LSM ± SEM; HS = effect of health status; Wk = effect of week); Supplementary Figure S6: Concentrations of protein in the milk of cows with (A) metritis, (B) retained placenta, (C) ketosis, (D) lameness, and (E) mastitis between healthy, low-disease, and high-disease (LSM ± SEM; HS = effect of health status; Wk = effect of week; Wk*P = effect of sampling week and parity interaction); Supplementary Figure S7: Concentrations of fat in the milk of cows with (A) metritis, (B) retained placenta, (C) ketosis, (D) lameness, and (E) mastitis between healthy, low-disease, and high-disease (LSM ± SEM; HS = effect of health status; Wk = effect of week); Supplementary Figure S8: Concentrations of milk urea nitrogen (Milk urea N) for (A) metritis, (B) retained placenta, (C) ketosis, (D) lameness, and (E) mastitis between healthy, low-disease, and high-disease (LSM ± SEM; HS = effect of health status; Wk = effect of week; Wk*P = effect of week * parity interaction); Supplementary Figure S9: Concentrations of total solids for (A) metritis, (B) retained placenta, (C) ketosis, (D) lameness, and (E) mastitis between healthy, low-disease, and high-disease (LSM ± SEM; HS = effect of health status; Wk = effect of week).

## References

[1] Roberson J.R. (2012). Treatment of Clinical Mastitis. Vet. Clin. North Am. Food Anim. Pract..

[2] Pitkala A., Haveri M., Pyorala S., Myllys V., Honkanen-Buzalski T. (2004). Bovine mastitis in Finland 2001-prevalence, distribution of bacteria, and antimicrobial resistance. J. Dairy Sci..

[3] Aghamohammadi M., Haine D., Kelton D.F., Barkema H.W., Hogeveen H., Keefe G.P., Dufour S. (2018). Herd-Level Mastitis-Associated Costs on Canadian Dairy Farms. Front. Vet. Sci..

[4] Napel J.T., de Haas Y., de Jong G., Lam T.J.G.M., Ouweltjes W., Windig J.J. (2009). Characterization of distributions of somatic cell counts. J. Dairy Sci..

[5] Djabri B., Bareille N., Beaudeau F., Seegers H. (2002). Quarter milk somatic cell count in infected dairy cows: A meta-analysis. Vet. Res..

[6] Madouasse A., Huxley J.N., Browne W.J., Bradley A.J., Green M.J. (2010). Somatic cell count dynamics in a large sample of dairy herds in England and Wales. Prev. Vet. Med..

[7] Shuster D.E.R.J., Harmon J.A., Jackson R.E., Hemken R.W. (1991). Suppression of Milk Production During Endotoxin-Induced Mastitis. J. Dairy Sci..

[8] Auldist M.J., Hubble I.B. (1998). Effects of mastitis on raw milk and dairy products. Aust. J. Dairy Technol..

[9] LeBlanc S.J., Duffield T.F., Leslie K.E., Bateman K.G., Keefe G.P., Walton J.S., Johnson W.H. (2002). Defining and diagnosing postpartum clinical endometritis and its impact on reproductive performance in dairy cows. J. Dairy Sci..

[10] Dervishi E., Zhang G., Hailemariam D., Dunn S.M., Ametaj B.N. (2016). Occurrence of retained placenta is preceded by an inflammatory state and alterations of energy metabolism in transition dairy cows. J. Anim. Sci. Biotechnol..

[11] Nocek J.K. (1997). Bovine acidosis: Implications for laminitis. J. Dairy Sci..

[12] Gordon J.L., LeBlanc S.J., Duffield T.F. (2013). Ketosis treatment in lactating dairy cattle. Vet. Clin. N. Am. Food Anim. Pract..

[13] Opdebeeck J.P. (1982). Mammary gland immunity. J. Am. Vet. Med. Assoc..

[14] Holdaway R.J. (1990). A Comparison of Methods for the Diagnosis of Bovine Subclinical Mastitis within New Zealand Dairy Herds. Ph.D. Thesis.

[15] Auldist M.J., Coats S., Rogers G.L., McDowell G.H. (1995). Changes in the composition of milk from healthy and mastitic dairy cows during the lactation cycle. Aus. J. Exp. Agr..

[16] Sjaastad Ø.V., Sand O., Hove K. (2016). Physiology of Domestic Animals.

[17] Dingwell R.T., Kelton D.F., Leslie K.E., Edge V.L. Deciding to dry-off: Does level of production matter?. Proceedings of the Annual Meeting of National Mastitis Council, NMC.

[18] Smith K.L., Todhunter D.A., Schoenberger P.S. (1985). Environmental mastitis: Cause, prevalence, prevention. J. Dairy Sci..

[19] Arnold M. (2012). Management of the Dry Cow to Prevent Mastitis.

[20] Goff J.P., Horst R.L. (1997). Physiological changes at parturition and their relationship to metabolic disorders. J. Dairy Sci..

[21] Mallard B.A., Dekkers J.C., Ireland M.J., Leslie K.E., Sharif S., Vankampen C.L., Wagter L., Wilkie B.N. (1998). Alteration in immune responsiveness during the peripartum period and its ramification on dairy cow and calf health. J. Dairy Sci..

[22] Ametaj B.N., Bradford B.J., Bobe G., Nafikov R.A., Lu Y., Young J.W., Beitz D.C. (2005). Strong relationships between mediators of the acute phase response and fatty liver in dairy cows. Can. J. Anim. Sci..

[23] Opsomer G. (2015). Metritis and endometritis in high yielding dairy cows. Rev. Bras. Reprod. Anim. Belo Hor..

[24] Boosman R., Mutsaers C.W., Klarenbeek A. (1991). The role of endotoxin in the pathogenesis of acute bovine laminitis. Vet. Quart..

[25] Archer S.C., Green M.J., Madouasse A., Huxley J.N. (2011). Association between somatic cell count and serial locomotion score assessments in UK dairy herds. J. Dairy Sci..

[26] Dervishi E., Zhang G., Hailemariam D., Dunn S.M., Ametaj B.N. (2015). Innate immunity and carbohydrate metabolism alterations precede occurrence of subclinical mastitis in transition dairy cows. J. Anim. Sci. Technol..

[27] Ametaj B.N., Zebeli Q., Iqbal S. (2010). Nutrition, microbiota, and endotoxin-related diseases in dairy cows. Rev. Brasil. Zootec..

[28] Eckel E.F., Ametaj B.N. (2016). Invited Review: Roles of bacterial endotoxins in the etiopathogenesis of periparturient diseases of transition dairy cows. J. Dairy Sci..

[29] Hakogi E., Tamura H., Tanaka S., Kohata A., Shimada Y., Tabuchi K. (1989). Endotoxin levels in milk and plasma of mastitis-affected cow measured with a chromogenic limulus text. Vet. Microbiol..

[30] Dosogne H., Meyer E., Struk A., van Loon J., Massart-Leen A.M., Burvenich C. (2002). Effect of enrofloxacin treatment of plasma endotoxin during bovine *Escherichia coli* mastitis. Inflam. Res..

[31] Canadian Council on Animal Care (1993). Guide to the Care and Use of Experimental Animals. http://www.ccac.ca.

[32] Hutjens M. (1999). Evaluation of Manure on the Farm. Illinois Livestock Trial by University of Illinois. http://livestocktrail.illinois.edu/dairynet/paperDisplay.cfm?ContentID=550.

[33] Sprecher D.J., Hostetler D.E., Kaneene J.B. (1997). Locomotion Scoring of Dairy Cattle. Theriogenology.

[34] (2018). MedCalc Statistical Software.

[35] Zhang G., Hailemariam D., Dervishi E., Goldansaz S.A., Deng Q., Dunn S.M., Ametaj B.N. (2016). Dairy cows affected by ketosis shown alterations in innate immunity and lipid and carbohydrate metabolism during the dry off period and postpartum. Res. Vet. Sci..

[36] Abuajamieh M., Kvidera S.K., Fernandez M.V., Nayeri A., Upah N.C., Nolan E.A., Lei S.M., DeFrain J.M., Green H.B., Schoenberg K.M. (2016). Inflammatory biomarkers are associated with ketosis in periparturient Holstein cows. Res. Vet. Sci..

[37] Zebeli Q., Sivaraman S., Dunn S.M., Ametaj B.N. (2011). Intermittent parenteral administration of endotoxin triggers metabolic and immunological alterations typically associated with displaced abomasum and retained placenta in periparturient dairy cows. J. Dairy Sci..

[38] Työppönen J., Kauppinen K. (1980). The stability and automatic determination of ketone bodies in blood samples taken in field conditions. Acta Vet. Scand..

[39] Kelley N., Jeltema D., Duan Y., He Y. (2019). The NLRP3 Inflammasome: An overview of mechanisms of activation and regulation. Int. J. Mol. Sci..

[40] Bergsten C. (2003). Causes, risk factors, and prevention of laminitis and related claw lesions. Acta Vet. Scand..

[41] Parker L.C., Jones E.C., Prince L.R., Dower S.K., Whyte M.K., Sabroe I. (2005). Endotoxin tolerance induces selective alterations in neutrophil function. J. Leukoc. Biol..

[42] Lavon Y., Leitner G., Moallem U., Klipper E., Voet H., Jacoby S., Glick G., Meidan R., Wolfenson D. (2011). Immediate and carryover effects of Gram-negative and Gram-positive toxin-induced mastitis on follicular function in dairy cows. Theriogenology.

[43] Battaglia D.F., Krasa H.B., Padmanabhan V., Viguie C., Karsch F.J. (2000). Endocrine alterations that underlie endotoxin-induced disruption of the follicular phase in ewes. Biol. Repro..

[44] Zhang G., Hailemariam D., Dervishi E., Deng Q., Goldansaz S.A., Dunn S.M., Ametaj B.N. (2015). Alterations of innate immunity reactants in transition dairy cows before clinical signs of lameness. Animals.

[45] Dervishi E., Zhang G., Hailemariam D., Goldansaz S.A., Deng Q., Dunn S.M., Ametaj B.N. (2016). Alterations in innate immunity reactants and carbohydrate and lipid metabolism precede occurrence of metritis in transition dairy cows. Res. Vet. Sci..

[46] Smith K.L., Oliver S.P. (1981). Lactoferrin: A component of nonspecific defense of the involuting bovine mammary gland. Adv. Exp. Med. Biol..

[47] Persson W.K., Bengtsson B., Lindberg A., Nyman A., Unnerstad E.H. (2009). Incidence of mastitis and bacterial findings at clinical mastitis in Swedish primiparous cow-influence of breed and stage of lactation. Vet. Microbiol..

[48] Cucarella C., Tormo M.A., Ubeda C., Trotonda M.P., Monzón M., Peris C., Amorena B., Lasa Í., Penadés J.R. (2004). Role of biofilm-associated protein bap in the pathogenesis of bovine *Staphylococcus aureus*. Infect. Immun..

[49] Cook N.B. (2003). Prevalence of lameness among dairy cattle in Wisconsin as a function of housing type and stall surface. J. Am. Vet. Med. Assoc..

[50] McManaman J.L., Neville M.C. (2003). Mammary physiology and milk secretion. Adv. Drug Deliv. Rev..

[51] Bruckmaier R.M., Ontsouka C.E., Blum J.W. (2004). Fractionized milk composition in dairy cows with subclinical mastitis. Vet. Med. Czech.

[52] Kobayashi K., Kuki C., Oyama S., Kumura H. (2016). Pro-inflammatory cytokine TNF-α is a key inhibitor for lactose synthesis pathway in lactating mammary epithelial cells. Exp. Cell Res..

[53] Boehmer J.L., Ward J.L., Peters R.R., Shefcheck K.J., McFarland M.A., Bannerman D.D. (2010). Proteomic analysis of the temporal expression of bovine milk proteins during coliform mastitis and label-free quantification. J. Dairy Sci..

[54] Shea-eaton W.K., Lee P.P.H., Ip M.M. (2001). Regulation of milk protein gene expression in normal mammary epithelial cells by tumor necrosis factor. Endocrinology.

[55] National Mastitis Council (1998). Current Concepts of Bovine Mastitis.

[56] Ip M.M., Shoemaker S.F., Darcy K.M. (1992). Regulation of rat mammary epithelial cell proliferation and differentiation by tumor necrosis factor-alpha. Endocrinology.

[57] Fahey J. (2008). Milk protein percentage and dairy cow fertility. Univ. Melb. Dep. Vet. Sci. VIAS Sneydes Road.

[58] Çağdaş C. (2013). Physiological and metabolic changes during the transition period and the use of calcium propionate for prevention or treatment of hypocalcemia and ketosis in periparturient cows. J. Biol. Environ. Sci..

[59] Sordillo L.M., Shafer-Weaver K., DeRosa D. (1997). Symposium: Bovine immunology. Immunology of the mammary gland. J. Dairy Sci..

[60] Ametaj B.N., Hosseini A., Odhiambo J.F., Iqbal S., Deng Q., Lam T.H., Farooq U., Zebli Q., Dunn S.M., Veas F. (2011). Application of acute phase proteins for monitoring inflammatory states in cattle. Acute Phase Proteins as Early Non-Specific Biomarkers of Human and Veterinary Diseases.

[61] Minuti A., Zhou Z., Graugnard D.E., Rodriguez-Zas S.L., Palladino A.R., Cardoso F.C., Trevisi E., Loor J.J. (2015). Acute mammary and liver transcriptome responses after an intramammary *Escherichia coli* lipopolysaccharide challenge in postpartal dairy cows. Physiol. Rep..

[62] Heuer C., Schukken Y.H., Dobbelaar P. (1999). Postpartum body condition score and results from the first test day milk as predictors of disease, fertility, yield, and culling in commercial dairy herds. J. Dary Sci..

[63] Toni F., Vincenti L., Grigoletto L., Ricci A., Schukken Y.H. (2011). Early lactation ratio of fat and protein percentage in milk is associated with health, milk production, and survival. J. Dary Sci..

[64] Petrovski K.R., Stefanov E. (2006). Milk Composition Changes during Mastitis.

[65] Turnbull A., Rivier C. (1999). Regulation of the hypothalamic-pituitary-adrenal axis by cytokines: Actions and mechanisms of action. Physiol. Rev..

