# Supplementary material for: Association of High Somatic Cell Counts Prior to Dry off to the Incidence of Periparturient Diseases in Holstein Dairy Cows
Source: Vet Sci. 2022 Nov 9;9(11):624. doi: 10.3390/vetsci9110624 (PMC9693442; doi:10.3390/vetsci9110624)
Supplement: Supplementary file 1 [file vetsci-09-00624-s001.zip › vetsci-1915077-supplementary.pdf]

Supplementary Material

Table S1. Frequency of cows diagnosed with disease for parity

Disease	Parity	
	Primiparous	Multiparous
Metritis	22 (37.3)	37 (62.7)
Retained Placenta	8 (40.0)	12 (60.0)
Mastitis	13 (31.7)	28 (68.3)
Ketosis	11 (24.4)	34 (75.6)
Laminitis	4 (18.2)	18 (81.8)
Healthy	22 (71.0)	9 (29.0)

Supplementary milk composition data

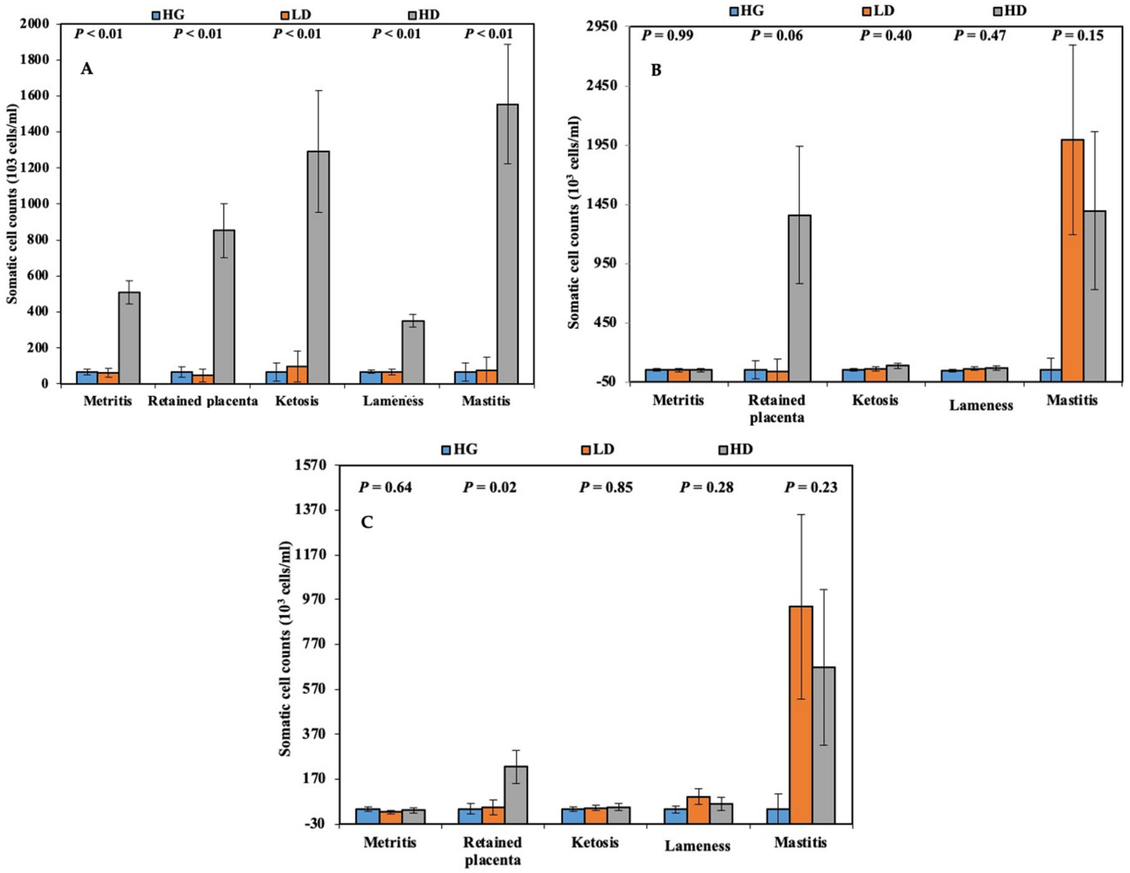

Figure S1. Comparisons of somatic cell counts (10^3 cells/mL) between healthy group (HG, blue), low disease (LD, orange), and high disease (HD, grey) for cows diagnosed with metritis, retained placenta, ketosis, lameness, and mastitis at A) Prior to dry off (~1 week before date of dry off); B) 1 week after parturition; C) 2 weeks after parturition.

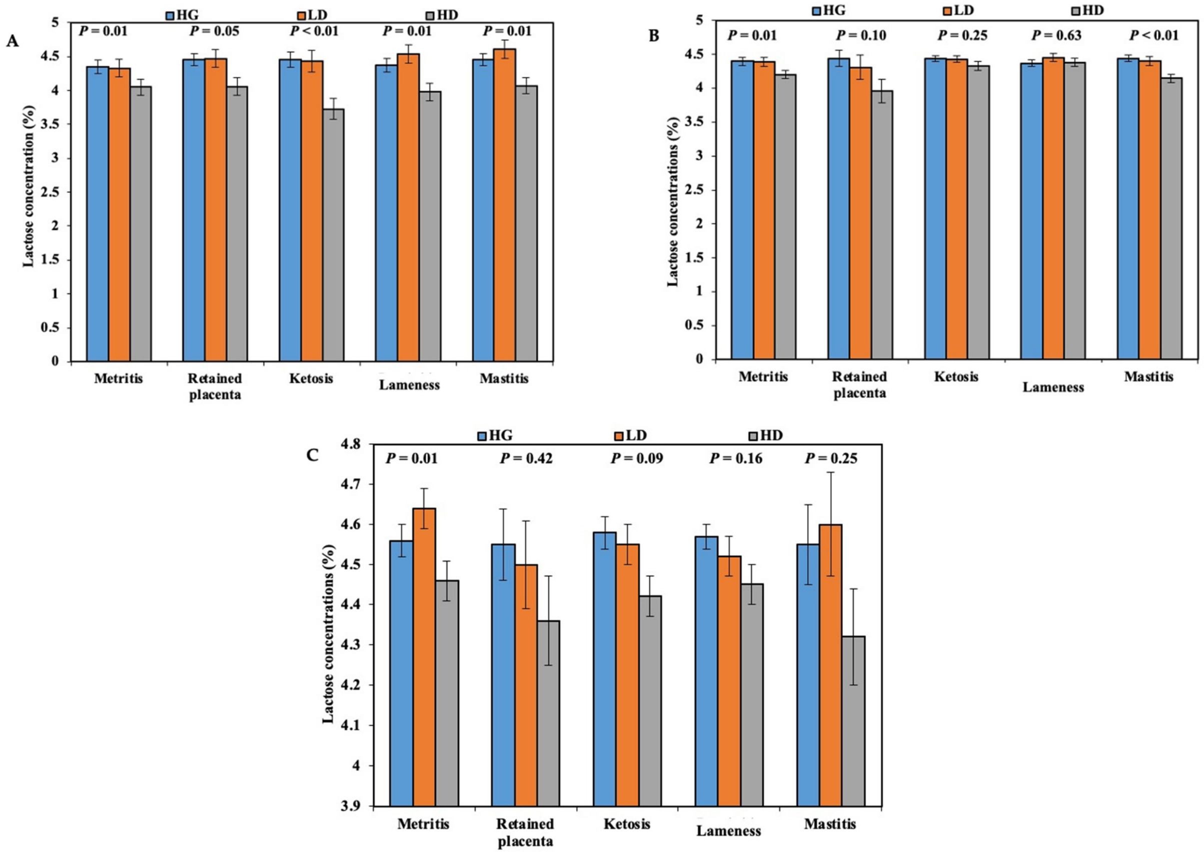

Figure S2. Comparisons of lactose concentrations (%) between healthy group (HG, blue bar), low disease (LD, orange bar), and high disease (HD, grey bar) for cows diagnosed with metritis, retained placenta, ketosis, lameness, and mastitis at A) Prior to dry off (~1 week before date of dry off); B) 1 weeks after parturition; C) 2 weeks after parturition.

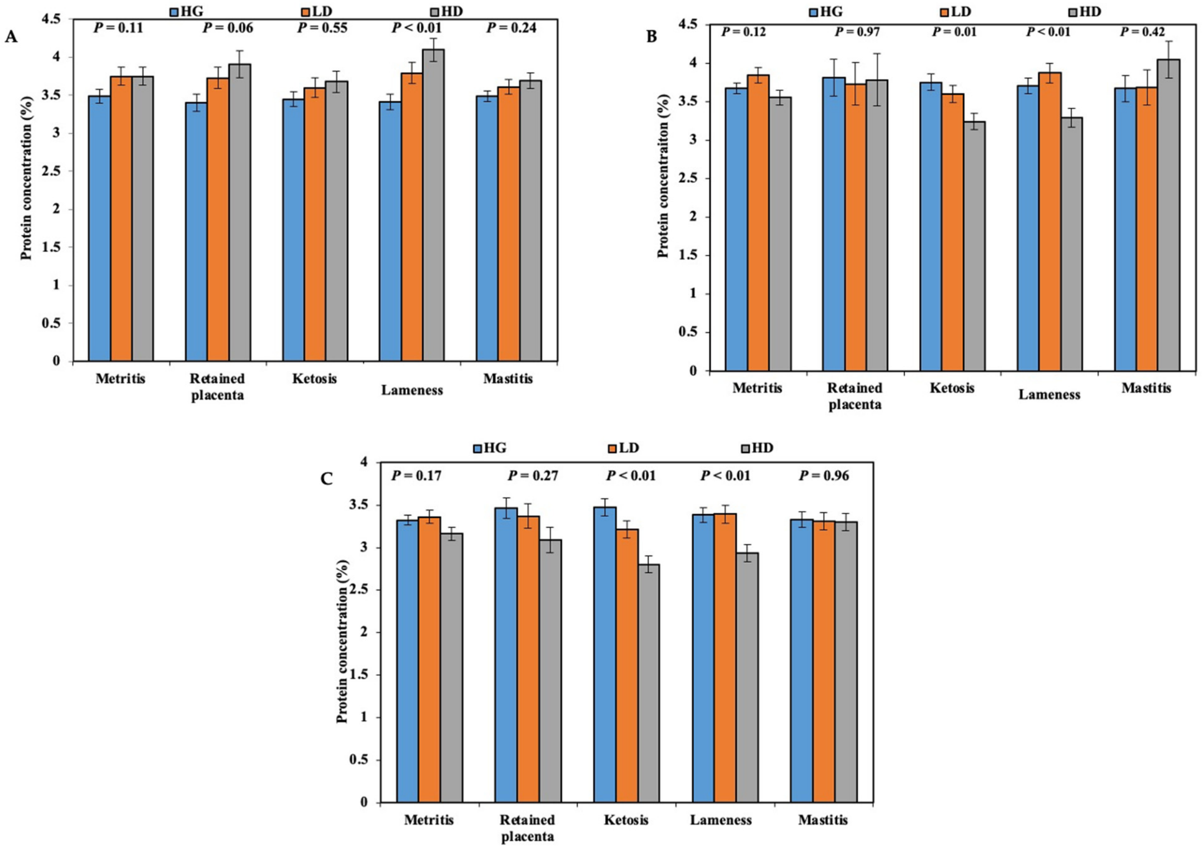

Figure S3. Comparisons of protein concentrations (%) between healthy group (HG, blue bar), low disease (LD, orange bar), and high disease (HD, grey bar) for cows diagnosed with metritis, retained placenta, ketosis, lameness, and mastitis at A) Prior to dry off (~1 week before date of dry off); B) 1 weeks after parturition; C) 2 weeks after parturition.

Milk composition data throughout experimental period

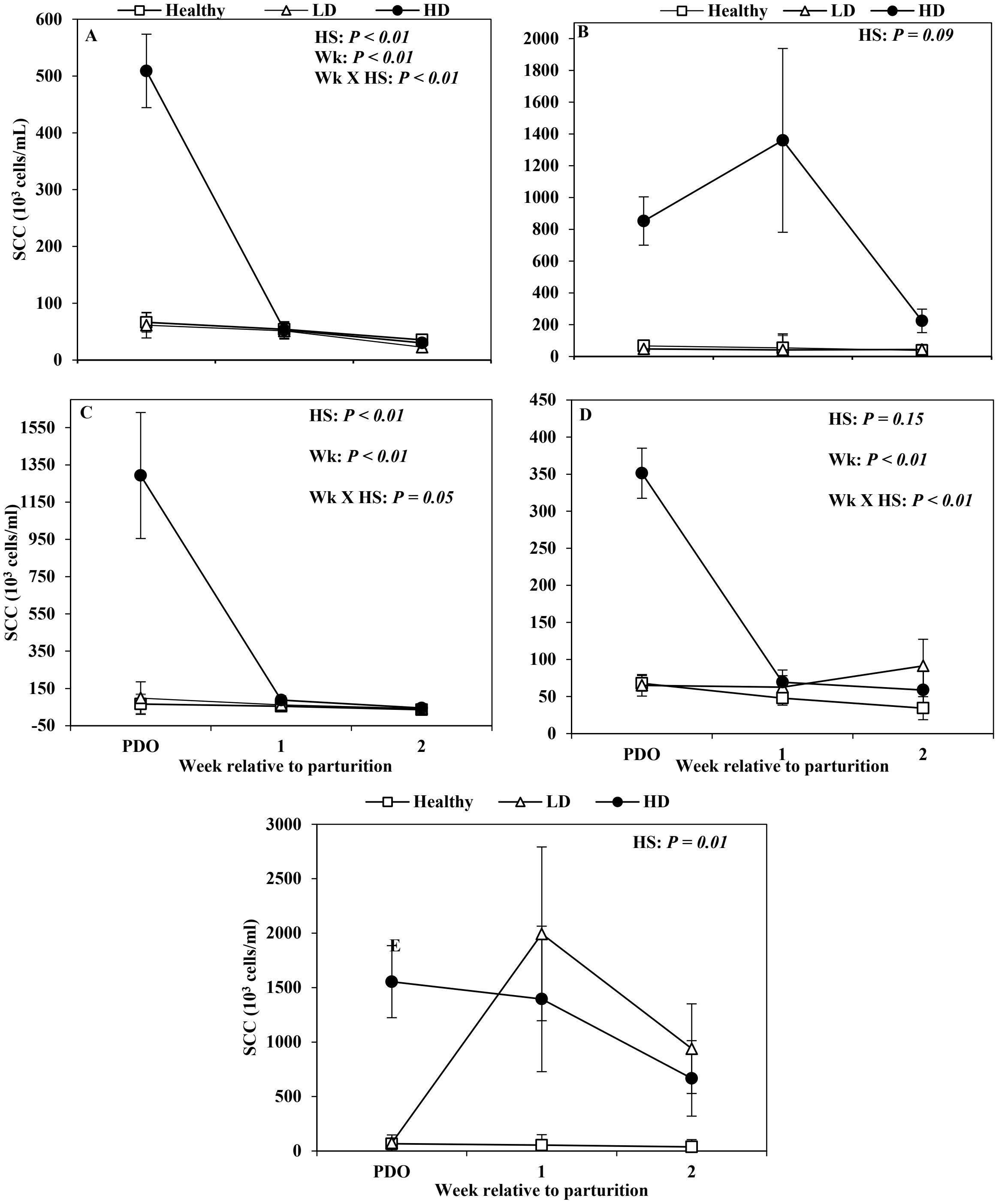

Figure S4. Concentrations of somatic cell counts with A) metritis, B) retained placenta, C) ketosis, D) lameness, and E) mastitis between healthy, low-disease, and high-disease (LSM±SEM; HS = effect of health status; Wk = effect of week; Wk x HS = week x health status interaction).

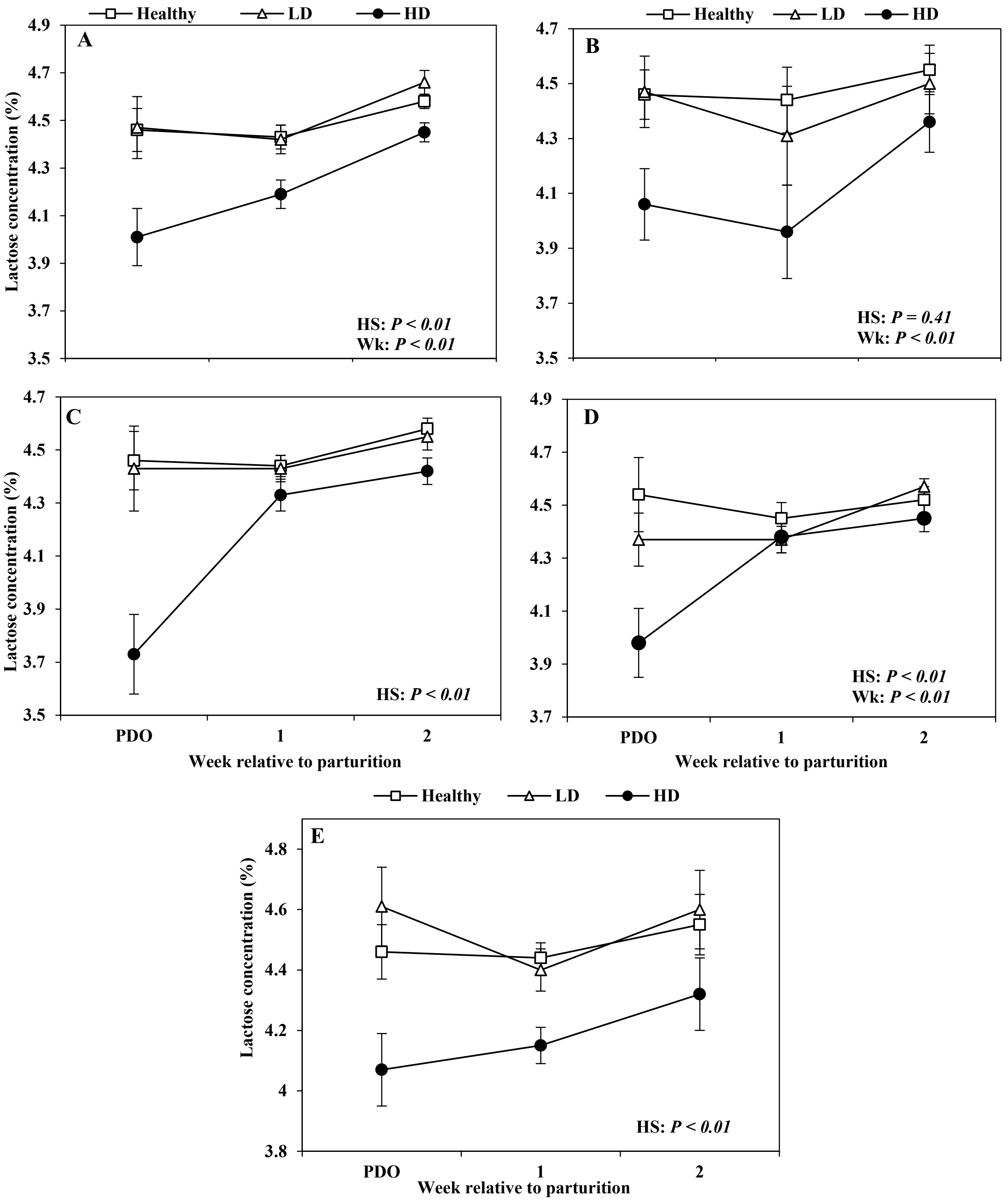

Figure S5. Concentrations of lactose in the milk of cows with A) metritis, B) retained placenta, C) ketosis, D) lameness, and E) mastitis between healthy, low-disease, and high-disease (LSM±SEM; HS = effect of health status; Wk = effect of week).

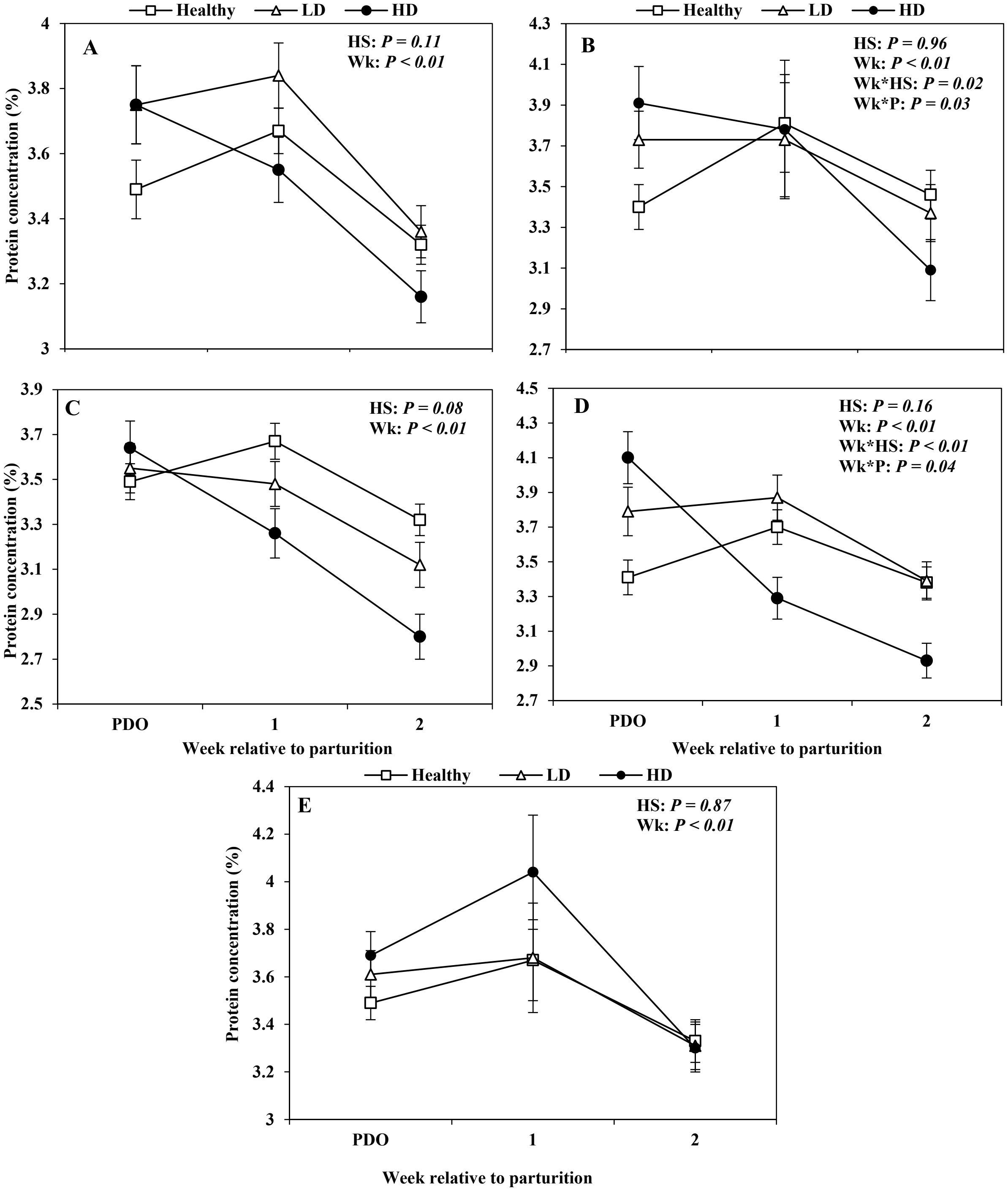

Figure S6. Concentrations of protein in the milk of cows with A) metritis, B) retained placenta, C) ketosis, D) lameness, and E) mastitis between healthy, low-disease, and high-disease (LSM±SEM; HS = effect of health status; Wk = effect of week; Wk*P = effect of sampling week and parity interaction).

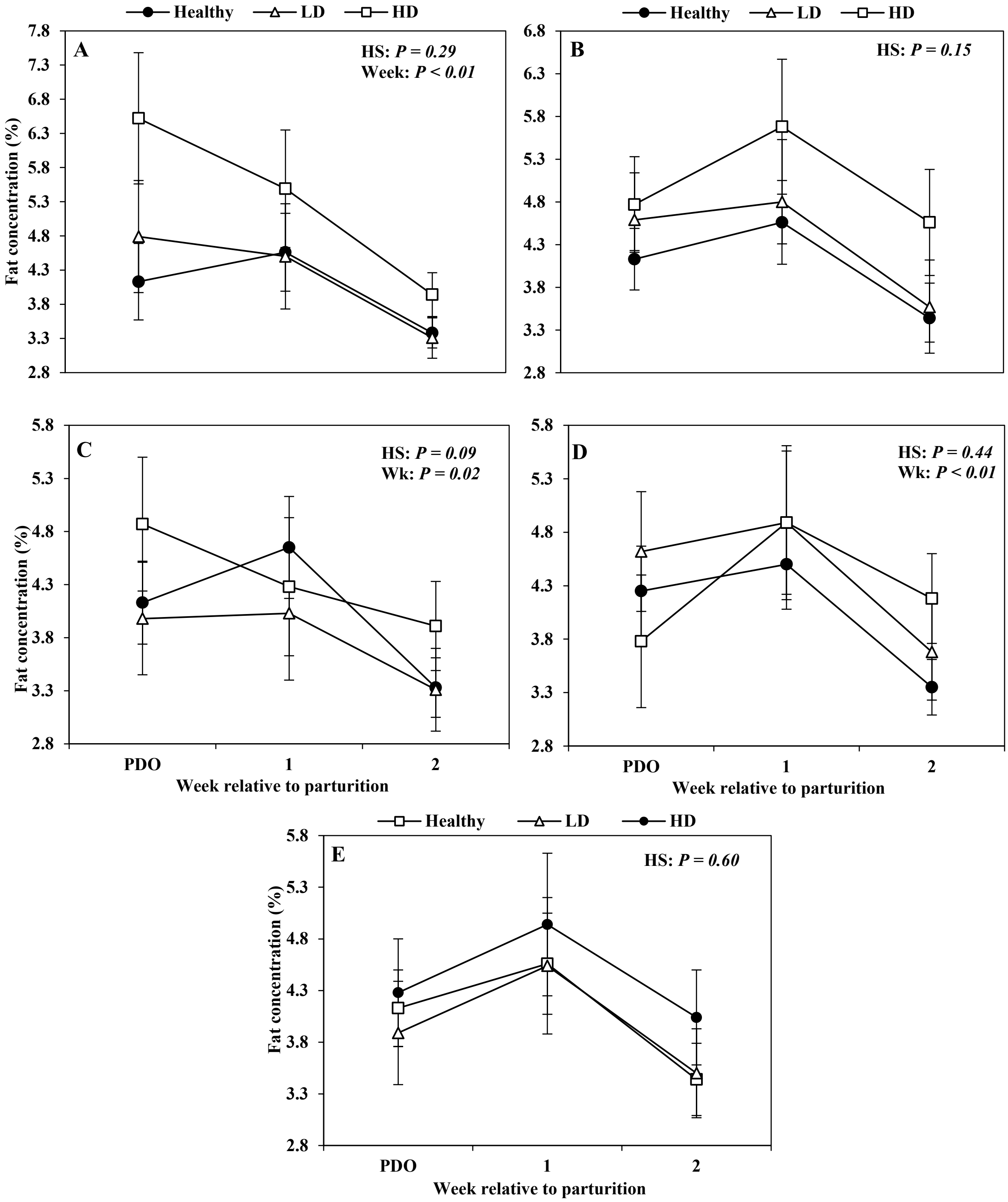

Figure S7. Concentrations of fat in the milk of cows with a) metritis, B) retained placenta, C) ketosis, D) lameness, and E) mastitis between healthy, low-disease, and high-disease (LSM±SEM; HS = effect of health status; Wk = effect of week).

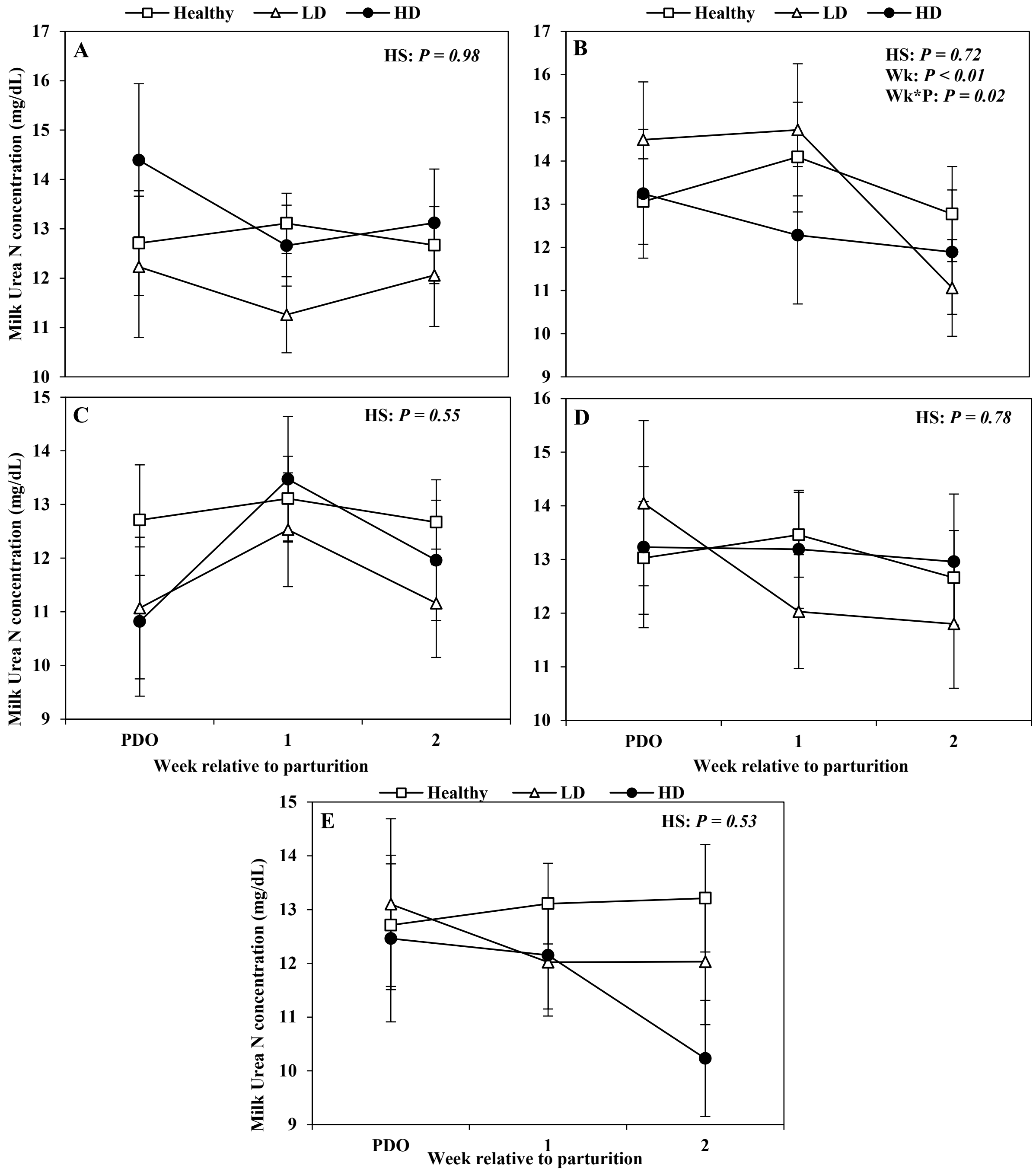

Figure S8. Concentrations of milk urea nitrogen (Milk urea N) for a) metritis, B) retained placenta, C) ketosis, D) lameness, and E) mastitis between healthy, low-disease, and high-disease (LSM±SEM; HS = effect of health status; Wk = effect of week; Wk*P = effect of week * parity interaction).

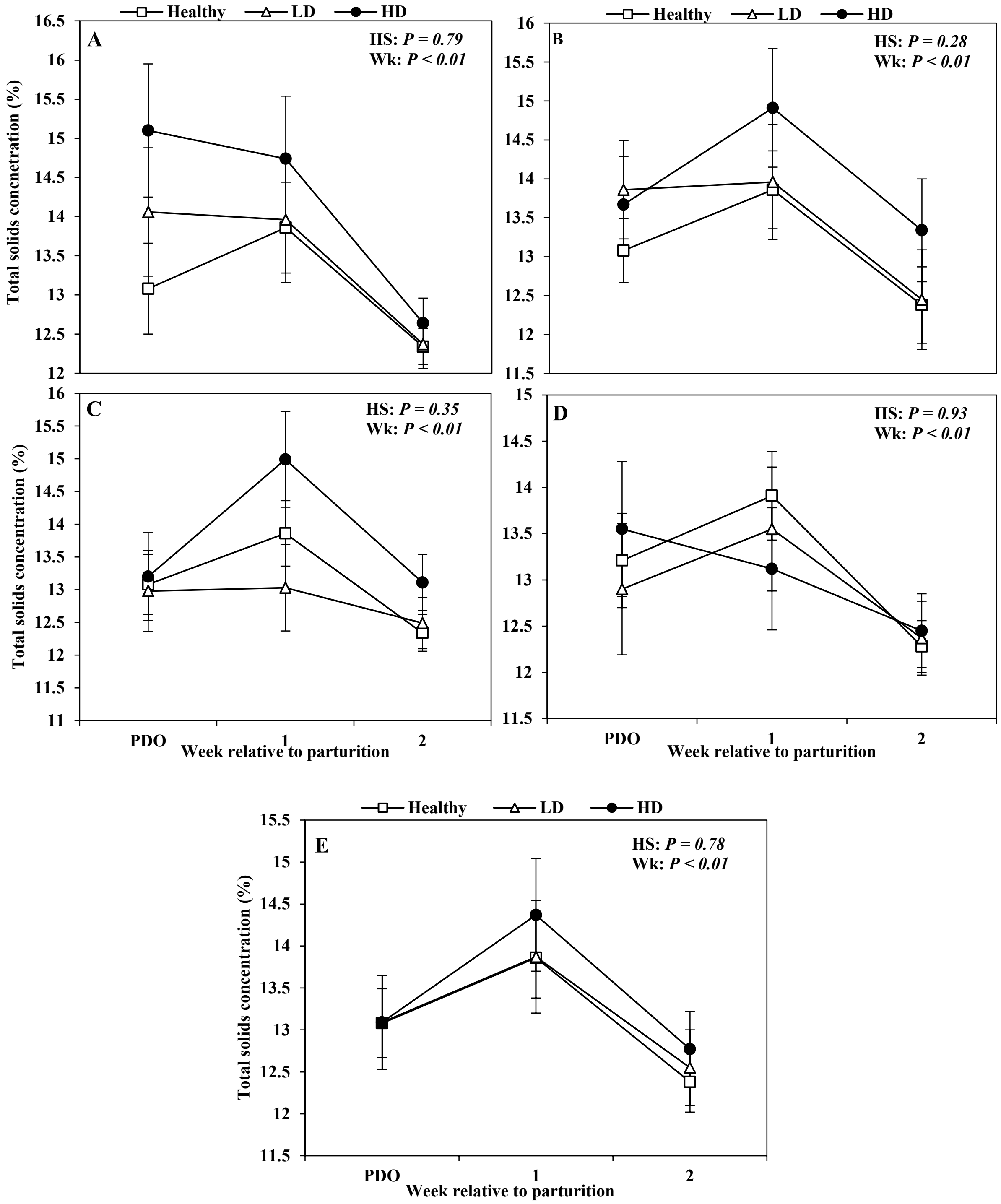

Figure S9. Concentrations of total solids for a) metritis, B) retained placenta, C) ketosis, D) lameness, and E) mastitis between healthy, low-disease, and high-disease (LSM±SEM; HS = effect of health status; Wk = effect of week).
